# E12.5 pancreatic bud-derived cells are detected in recipient pancreata after intraplacental transplantation, but *Pdx1*-deficient recipients lack a macroscopically detectable pancreas

**DOI:** 10.1038/s41598-026-65314-w

**Published:** 2026-09-14

**Authors:** Zeynab Javanfekr Shahri, Atsushi Noda, Takuto Hayashi, Ching-Wei Liao, Natalia Gogoleva, Arata Wakimoto, Eugenia Kumaga, Michito Hamada, Satoru Takahashi

**Affiliations:** 1 Department of Anatomy and Embryology, Faculty of Medicine, University of Tsukuba, 1‐1‐1, Tennodai, Tsukuba, Ibaraki 305‐8575 Japan; 2 Ph.D. Program in Human Biology, School of Integrative and Global Majors, University of Tsukuba, 1‐1‐1, Tennodai, Tsukuba, Ibaraki 305-8575 Japan; 3 Université Paris Cité, INSERM UMR-S1151, CNRS UMR-S8253, Institut Necker Enfants Malades, Paris, France; 4 Department of Obstetrics and Gynecology, School of Medicine, University of Washington, Seattle, WA 98109 USA; 5 Laboratory Animal Resource Center, Faculty of Medicine, University of Tsukuba, 1‐1‐1, Tennodai, Tsukuba, Ibaraki 305‐8575 Japan; 6 Center for Medical Sciences, Ibaraki Prefectural University of Health Sciences, 4669-2 Ami, Inashiki-gun, Ami-machi, Ibaraki Japan

**Keywords:** Pancreatic organogenesis, Intraplacental transplantation, Pancreatic bud-derived cells, *Pdx1*-deficient mice, Limited endocrine/metabolic signal, *Epcam*, Cell biology, Developmental biology, Stem cells

## Abstract

The behavior of embryonic pancreatic bud-derived cells after intraplacental transplantation (IPT) into non-knockout versus *Pdx1*-deficient embryonic recipient contexts remains unclear. Here, we used IPT to compare the fate of dissociated E12.5 mouse pancreatic bud-derived cells in non-knockout recipients retaining an endogenous pancreatic developmental field and *Pdx1*-deficient mouse recipients. In a subset of non-knockout hosts, donor-derived cells were detected within endocrine, acinar, and ductal compartments, indicating donor-cell localization within pancreatic tissue compartments after transplantation. In contrast, grossly examined *Pdx1*-deficient recipients did not form macroscopically detectable organized pancreatic tissue. Some KO recipients in a serum-insulin endpoint cohort showed detectable circulating insulin. In a separate survival/blood-glucose cohort, IPT KO recipients showed transient postnatal increases in blood glucose followed by progressive hypoglycemia; survival and overall physiology were not normalized. Donor-cell localization in *Pdx1*-deficient recipients was not determined in this study. Thus, under the present donor-preparation and IPT conditions, E12.5 pancreatic bud-derived cells were detected within pancreatic tissue compartments in a subset of non-KO recipients but did not form macroscopically detectable organized pancreatic tissue in *Pdx1*-deficient recipients. Separately, analysis of published single-cell RNA-sequencing datasets, together with flow-cytometric validation, supported EPCAM as a reporter-independent epithelial enrichment marker that captures a large fraction of *Pdx1*-expressing epithelial cells in the E12.5 pancreatic bud. These parallel findings show that transplantation outcome differed between non-KO recipients retaining an endogenous pancreatic field and *Pdx1*-deficient recipients under the present IPT conditions and also support a practical strategy for enriching embryonic pancreatic epithelial fractions containing *Pdx1*-expressing cells.

**Supplementary Information:**

The online version contains supplementary material available at https://doi.org/10.1038/s41598-026-65314-w.

## Introduction

Stem cell–based replacement strategies have advanced substantially, including protocols that generate glucose-responsive insulin-secreting β-like cells from human pluripotent stem cells and improve glycemic control after transplantation^1,2^. Loss of functional β-cell mass remains a major challenge in type 1 diabetes and regenerative medicine^3–5^. However, durable replacement of pancreatic function requires more than endocrine hormone production alone. Reconstruction of organized pancreatic tissue may additionally require coordinated epithelial morphogenesis, lineage diversification, and integration within an appropriate developmental tissue context^5–8^.

In this context, it is important to distinguish limited endocrine/metabolic signals after transplantation from organized pancreatic tissue formation.

PDX1 (pancreatic and duodenal homeobox 1) is a key regulator of pancreatic development and β-cell identity. During embryogenesis, *Pdx1* is expressed in pancreatic progenitor populations and is required for pancreatic outgrowth and lineage specification. In mice, loss of *Pdx1* causes pancreatic agenesis and neonatal lethality, establishing the *Pdx1*-null model as a classical system for studying failed pancreatic organogenesis^9^. Consistent with this, studies of pancreatic developmental biology place *Pdx1* within the core transcriptional network governing progenitor maintenance and endocrine differentiation^10^. In humans, pathogenic *PDX1* variants are also associated with pancreatic developmental defects and monogenic diabetes, supporting a conserved role across species^11^.

We previously developed an intraplacental transplantation (IPT) approach that enables delivery of donor progenitor cells into the embryonic circulation during early gestation. Using this system, transplantation of human iPSC-derived PDX1^+^ pancreatic progenitors into *Pdx1*-deficient embryos prolonged neonatal survival and resulted in detectable insulin production, indicating that transplanted cells could undergo endocrine differentiation in vivo. However, donor-derived cells did not form an organized pancreas and instead localized predominantly within the duodenal wall^12^. These observations raised a central unresolved question: does the absence of organized pancreatic tissue formation reflect limitations of donor-cell state, or does it reflect recipient-side factors in the *Pdx1*-deficient setting that prevent organized pancreatic tissue assembly?

Discriminating between these possibilities is important for both developmental biology and regenerative medicine. If organogenesis failure primarily reflects donor immaturity or lineage restriction, then improving donor differentiation state may be sufficient to improve pancreatic tissue formation. In contrast, if recipient context, donor-cell delivery, survival, localization, or timing is limiting, then even embryonic pancreatic bud-derived cells may fail to generate organized pancreatic tissue in a defective embryonic environment. These variables may influence transplantation outcome in IPT-based studies^12–14^.

*Emb*ryonic pancreatic buds provide an opportunity to address this problem using donor cells from a developmental stage with documented multilineage pancreatic potential. At E12.5, the mouse pancreatic epithelium contains heterogeneous progenitor and differentiating populations, including tip-biased acinar progenitor populations and trunk populations associated with ductal and endocrine development^15–18^. Single-cell transcriptomic studies further show that these embryonic epithelial populations express key developmental regulators, including *Pdx1*, *Sox9*, *Hnf1b*, and *Nkx6-1*, consistent with multilineage pancreatic potential at this stage^17^. Thus, E12.5 pancreatic buds provide a physiologically relevant donor source for testing whether the recipient context supports donor-cell detection within pancreatic tissue compartments.

Because the composition of the transplanted donor population may influence engraftment outcome^12–14^, characterizing the cellular heterogeneity of the E12.5 pancreatic bud and establishing strategies to enrich epithelial fractions containing *Pdx1*-expressing cells are directly relevant to interpreting transplantation results and improving future protocols. In parallel, prospective enrichment of epithelial fractions containing pancreatic progenitor cells remains a practical need for transplantation-based studies. Although genetic reporters are valuable experimental tools, enrichment based on endogenous cell-surface markers would provide a more broadly applicable strategy. Previous studies have associated epithelial markers such as *Epcam*, *Cdh1*, and *Cldn6* with embryonic pancreatic epithelial compartments, while also highlighting substantial epithelial heterogeneity during pancreas development^17,19,20^. In addition, the Cell Surface Protein Atlas offers a curated resource for prioritizing experimentally validated surface proteins for cell-isolation approaches^21^.

Here, we used IPT to transplant dissociated E12.5 mouse pancreatic bud-derived cells into embryonic recipients and compared outcomes between non-KO recipients retaining an endogenous pancreatic developmental field and *Pdx1*-deficient recipients. Because recipient genotype was unknown at transplantation, the donor source and IPT protocol were standardized before postnatal genotyping; however, animal-level delivery efficiency, viable cell dose, donor-cell survival, and localization were not directly measured. This design allowed us to ask whether the same transplantation protocol produced donor-cell detection within pancreatic compartments in non-KO recipients and macroscopically detectable organized pancreatic tissue or endocrine/metabolic signals in *Pdx1*-deficient recipients. In parallel, we integrated published single-cell RNA-sequencing datasets with experimental validation to identify a practical reporter-independent surface marker for enrichment of embryonic pancreatic epithelial fractions containing *Pdx1*-expressing cells.

## Results

### Validation of E12.5 pancreatic bud-region isolation

To confirm that donor tissue was isolated from the embryonic pancreatic bud region, pancreatic buds were microdissected from E12.5 embryos using established anatomical landmarks. At this stage, the dorsal pancreatic bud is located within the dorsal mesogastrium adjacent to the developing splenic primordium, whereas the ventral bud lies near the stomach–duodenum junction^6,22^. Using these landmarks, the region containing the stomach, pancreatic buds, and adjacent duodenum was collected from wild-type and *Pdx1*^+/−^ embryos. To validate donor tissue identity, we used *Pdx1* expression as a marker of the embryonic pancreatic bud epithelium. In *Pdx1*^LacZ/+^ embryos, in which LacZ reports endogenous *Pdx1* transcriptional activity^9^, β-galactosidase staining revealed robust signal in the dissected pancreatic region, whereas no staining was detected in anatomically matched tissues from wild-type littermates (Supplementary Fig. 1A). The broader stomach-duodenum region was exposed to define the anatomical landmarks, but the pancreatic bud region indicated by the dotted outline in Supplementary Fig. 1A was the region collected for donor-cell preparation and subsequent IPT.

To further confirm that the isolated tissue contained *Pdx1*-expressing cells at the single-cell level, independently microdissected pancreatic regions were dissociated and subjected to β-galactosidase staining. Consistent with the whole-mount analysis, dissociated samples from *Pdx1*^+/−^ embryos showed reporter-positive single cells, whereas wild-type controls remained negative (Supplementary Fig. 1B). Together, these results confirm accurate isolation of E12.5 pancreatic bud-region tissue containing *Pdx1*-expressing epithelial cells for subsequent transplantation experiments.

### E12.5 pancreatic bud-derived cells are detected in multiple pancreatic compartments in non-KO hosts

Having confirmed that our isolation strategy enriched pancreatic bud-region tissue containing *Pdx1*-expressing epithelial cells (Supplementary Fig. 1), we next asked whether these embryonic cells could survive, engraft, and become detectable within multiple pancreatic tissue compartments in non-KO recipients retaining an endogenous pancreatic developmental field.

**Fig. 1:**
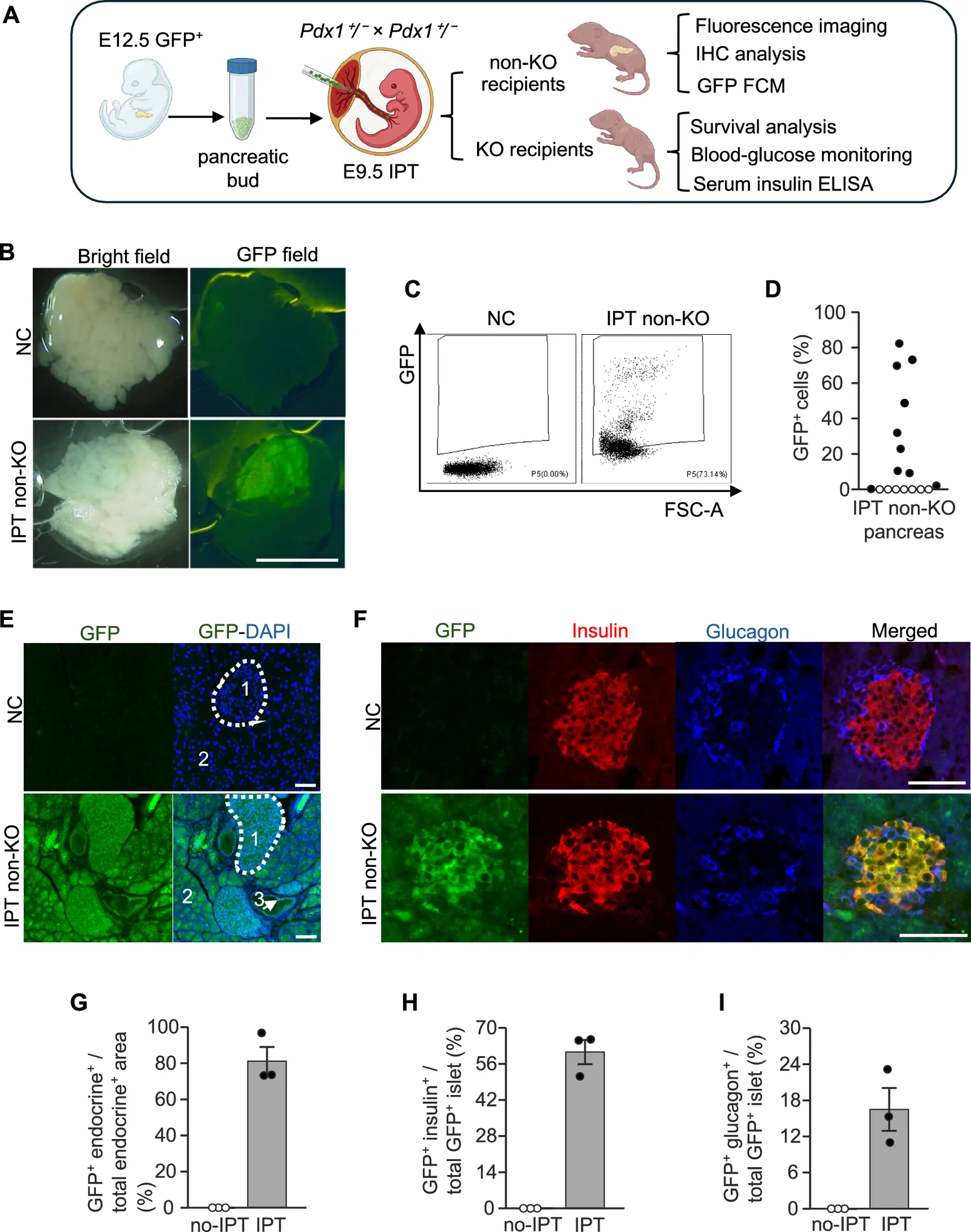
Experimental design and donor-cell detection across multiple pancreatic compartments in non-KO recipients. (**A**) Experimental overview of intraplacental transplantation (IPT). Pancreatic buds from E12.5 GFP^+^ donor embryos were dissociated into single-cell suspensions and transplanted intraplacentally into E9.5 embryos generated by *Pdx1*^+/−^ × *Pdx1*^+/−^ crosses. Recipients were genotyped postnatally and allocated to assay-specific cohorts. Panels B–I show non-KO recipients or recipient-derived samples. Panels E–I show the 2-month IHC cohort; GFP-positive pancreatic regions were detected in 5 of 8 recipients, and analyses in G–I were restricted to the three recipients with GFP-positive endocrine-islet signal. (**B**) Bright-field and GFP fluorescence stereomicroscopy of recipient pancreas at P1. Discrete GFP^+^ regions were detected in IPT recipients but not in non-transplanted controls (NC). Scale bar = 2 mm. (**C**) Representative flow-cytometry plots showing GFP^+^ donor-cell detection relative to an NC after sequential exclusion of debris, doublets, dead cells, and CD45^+^ cells. (**D**) GFP^+^ cell frequencies in P0–P3 pancreata from IPT non-KO recipients (n = 17). Each point represents one recipient. Open circles indicate GFP signal indistinguishable from background (n = 7); filled circles indicate GFP-positive donor cells detected above background (n = 10; 58.8%). No inferential comparison was performed because the categories were defined by the GFP-detection readout. (**E**) Representative pancreatic sections showing GFP (green) and DAPI (blue). Labels 1, 2, and 3 indicate endocrine islets, acinar tissue, and ducts, respectively. Scale bar = 50 μm. (**F**) Representative immunofluorescence for GFP (green), insulin (red), and glucagon (blue), showing donor-cell marker overlap with endocrine markers within islet structures. Scale bar = 50 μm. (**G**) GFP-positive percentage of the total endocrine-positive islet area, defined as insulin- and glucagon-positive area combined. (**H**) Insulin-positive percentage of the GFP-positive islet area. (**I**) Glucagon-positive percentage of the GFP-positive islet area. In G–I, bars show mean ± SEM and points represent the three recipients analyzed. No-IPT values are shown as GFP-background references; no statistical comparison was intended.

To enable donor-cell tracking, pancreatic buds were isolated from E12.5 GFP^+^ embryos, dissociated, and transplanted intraplacentally into E9.5 *Pdx1*^+/−^ × *Pdx1*^+/-^ embryos. Across all experiments, 113 embryos underwent intraplacental transplantation, of which 86 were born (76.1% birth rate). Genotyping of live-born pups identified 64 non-KO recipients (*Pdx1*^+/+^ or *Pdx1*^+/−^) and 22 KO recipients (*Pdx1*^−/−^), consistent with expected Mendelian ratios (Supplementary Table 1). All KO recipients were included in KO outcome analyses. Of the 64 live-born non-KO recipients, 32 contributed to downstream endpoint analyses summarized in Fig. 1A; detailed assay allocation is provided in Methods.

Fluorescence stereomicroscopy at postnatal day 1 (P1) in non-KO recipients revealed distinct GFP^+^ tissue within the pancreas (Fig. 1B). Because GFP expression was restricted to donor cells, these GFP^+^ structures indicate the presence of donor-derived cells within recipient pancreatic tissue.

Donor-cell detection was further quantified by flow cytometric analysis of pancreata collected between postnatal day 0 and postnatal day 3 (P0–P3). GFP^+^ cell frequencies were calculated after sequential exclusion of debris and doublets, dead cells, and CD45^+^ hematopoietic cells, with the full gating strategy shown in Supplementary Fig. 2A. GFP-positive events above background were detected in 58.8% of non-KO recipients (10/17), whereas 7 of 17 non-KO recipients showed GFP signal indistinguishable from background and no GFP^+^ cells were detected in non-transplanted controls (Fig. 1C, D). These endpoint data are presented descriptively as individual values and detection frequency because donor-cell-detected and non-detected categories were defined by the GFP detection readout itself. The variable donor-cell detection among recipients may reflect differences in effective placental delivery, vascular distribution, donor-cell survival, and access to the recipient pancreas^12–14^.

To assess longer-term donor-cell detection and compartmental localization, the IHC cohort of eight non-KO recipients was maintained for up to 2 months. Histological analysis detected GFP^+^ regions within host pancreatic tissue, including endocrine, acinar, and ductal compartments (Fig. 1E). Triple immunofluorescence staining showed overlap of GFP with insulin and glucagon within islet structures (Fig. 1F), consistent with donor-derived endocrine-marker expression. Quantitative islet-area analysis further assessed GFP-positive endocrine-marker overlap, including donor-derived endocrine-marker-positive area and insulin/glucagon co-localization within GFP-positive islet areas (Fig. 1G–I). Staining specificity was supported by GFP^+^ pancreas positive controls and no-primary-antibody controls (Supplementary Fig. 2B, C).

Among the eight IHC-analyzed non-KO recipients, GFP-positive pancreatic regions were detected in five animals, with variable compartmental distribution (Supplementary Fig. 3; Supplementary Table 2). Samples 1–3 showed GFP immunoreactivity in both endocrine islets and exocrine pancreatic compartments, whereas samples 4 and 5 showed exocrine GFP immunoreactivity without detectable GFP signal in endocrine islets. Samples 6–8 are not shown in Supplementary Fig. 3 because GFP-positive endocrine or exocrine pancreatic compartments were not detected; their endpoint classification is provided in Supplementary Table 2. These histological patterns were qualitatively consistent with the variable GFP-positive cell frequencies detected by flow cytometry in a separate neonatal cohort, but these assays were not designed to establish same-animal correlations.

Together, these results show that E12.5 pancreatic bud-derived donor cells were detected in endocrine, acinar, and ductal compartments, consistent with donor-cell marker expression and localization within host pancreatic tissue compartments.

### *Pdx1*-deficient recipients lack macroscopically detectable organized pancreatic tissue after IPT

We next examined KO recipients to determine whether the standardized donor source and IPT protocol would be associated with detectable endocrine/metabolic signals or organized pancreatic tissue formation in *Pdx1*^−/−^ recipients. Because recipient genotype was unknown at transplantation, donor source and IPT procedure were standardized before postnatal genotyping; however, this design did not directly measure animal-level donor-cell delivery efficiency, survival, localization, or viable cell dose. All live-born KO recipients (n = 22) were included in KO analyses and allocated to assay-specific cohorts: survival/blood-glucose assessment (n = 15) or serum insulin measurement by ELISA (n = 7) (Fig. 1A). The no-IPT KO controls were analyzed separately as survival/blood-glucose controls (n = 11) and serum-insulin negative controls (n = 3).

**Fig. 2:**
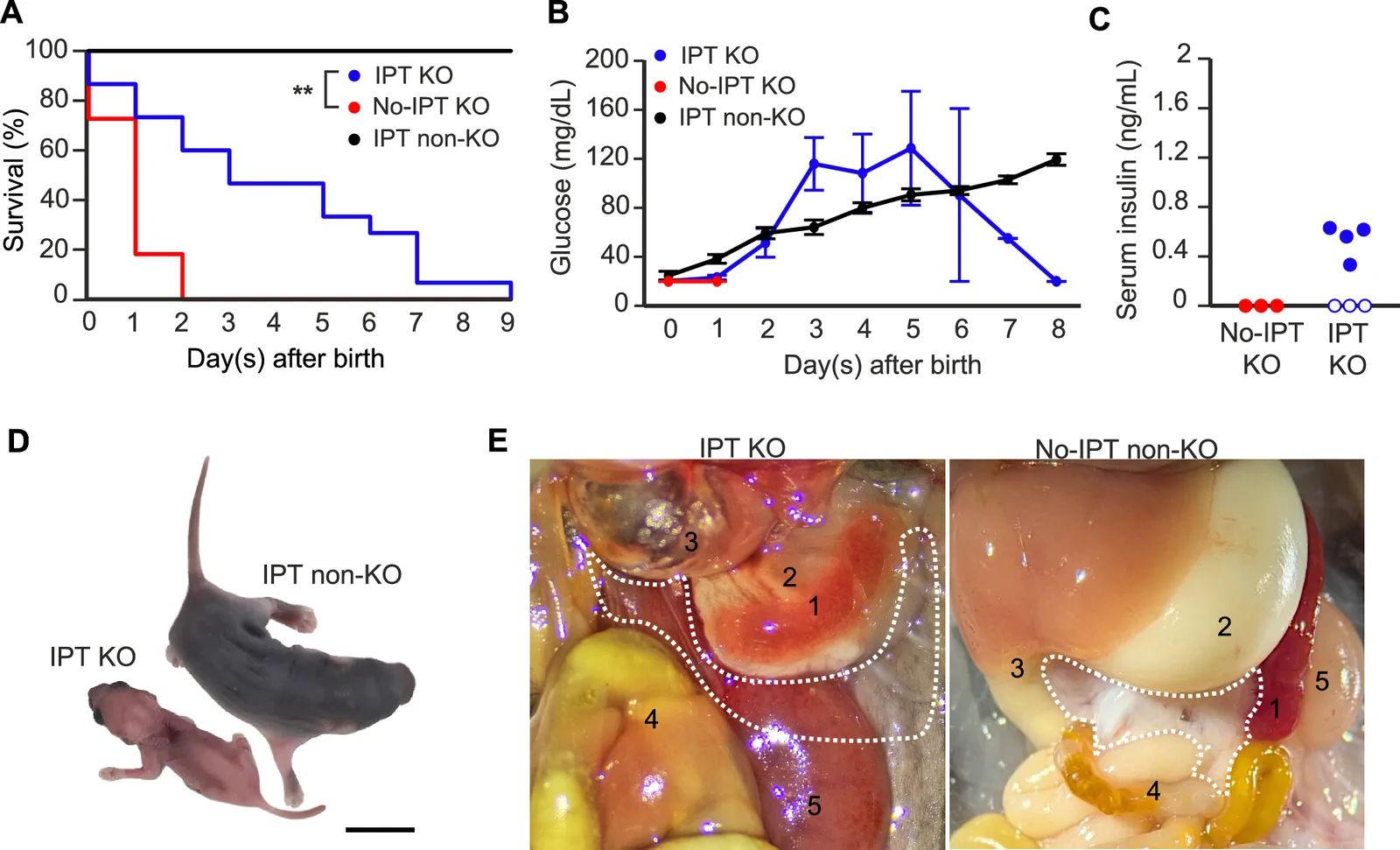
Limited endocrine/metabolic signals without macroscopically detectable organized pancreatic tissue in *Pdx1*-deficient recipients. (**A**) Kaplan–Meier survival curves for IPT KO neonates (n = 15), no-IPT KO controls (n = 11), and an IPT non-KO reference cohort (n = 7). IPT significantly prolonged KO survival relative to no-IPT KO controls (*P* = 0.0013, log-rank [Mantel–Cox] test). (**B**) Random blood-glucose trajectories from P0 until death in no-IPT KO (n = 11), IPT KO (n = 13), and IPT non-KO recipients (n = 7). Lines and shading show mean ± SEM. No-IPT KO recipients remained severely hypoglycemic, whereas IPT KO recipients showed transient increases followed by progressive hypoglycemia. Individual values are provided in Supplementary Table 3; animal numbers decreased over time because of KO mortality. Pups that died on P0 were included in survival analysis but excluded from blood-glucose analysis. (**C**) Serum insulin measured by ELISA in a separate endpoint cohort. Each point represents one recipient. All no-IPT KO controls were below the detection limit (n = 3; 0/3 detectable) and are shown as open red circles at zero. Among IPT KO recipients (n = 7), filled blue circles indicate detectable insulin (4/7), and open blue circles indicate values below the detection limit (3/7), plotted at zero. Samples were collected at available endpoints from P1 to P4; the exact sampling day for each sample was not recorded. No inferential comparison was performed because detectability groups were defined by the assay result. (**D**) Representative gross phenotype of an IPT KO recipient and an IPT non-KO littermate at P6. Scale bar = 1 cm. (**E**) Representative abdominal anatomy of an IPT KO recipient at P9 after death and an age-matched no-IPT non-KO recipient. Labels indicate spleen (1), stomach (2), duodenum (3), intestine (4), and kidney (5); the dashed outline marks the expected pancreatic region. No macroscopically detectable pancreatic structure was observed in the IPT KO recipient. Panels **D** and **E** are qualitative and were not used for group-wise morphometric analysis.

Transplanted *Pdx1*^−/−^ recipients (IPT KO) showed significantly prolonged survival relative to non-transplanted KO controls (Fig. 2A). Whereas non-transplanted KO mice (no-IPT KO) remained severely hypoglycemic throughout their short lifespan, with blood glucose persistently below 20 mg dL^−1^, transplanted KO recipients exhibited variable blood glucose trajectories during the early postnatal period (Fig. 2B). Some individuals showed transient postnatal increases in blood glucose; however, substantial inter-individual variability was observed, and blood glucose was not sustained in any recipient, with progressive decline to severe hypoglycemia preceding death. These blood-glucose data were obtained from the survival/blood-glucose cohort and were not matched to serum insulin measurements in the same animals. Individual blood-glucose measurements for the IPT KO, no-IPT KO, and IPT non-KO groups are provided in Supplementary Table 3.

Serum insulin was measured in a separate KO endpoint cohort by ELISA. Because *Pdx1*-deficient recipients lack a normal endogenous pancreatic insulin source^9^, detectable circulating insulin was considered consistent with a transplantation-associated endocrine/metabolic signal; however, the anatomical source and localization of insulin-producing cells were not determined. In the IPT KO endpoint cohort, 4 of 7 recipients had detectable serum insulin, whereas 3 of 7 did not; insulin was not detected in no-IPT KO negative controls (0/3) (Fig. 2C). The serum insulin cohort was separate from the survival/blood-glucose cohort; therefore, circulating insulin values could not be correlated with survival or blood-glucose trajectories in the same animals. These endpoint data are presented descriptively as individual values and detection frequencies because detectable-insulin status was defined by the assay result. This assay was used to confirm detectable circulating insulin rather than to compare regulated insulin secretory capacity; sampling details are provided in Methods.

At the cohort level, however, IPT-treated *Pdx1*^−/−^ recipients remained severely compromised. Grossly examined IPT KO neonates for which phenotypic records were available were markedly smaller than non-KO littermates, appeared dehydrated, and lacked visible subcutaneous adipose tissue (Fig. 2D). In grossly examined IPT KO recipients for which anatomical records were available, dissection revealed no macroscopically detectable organized pancreatic tissue in the examined anatomical region despite transplantation and gastrointestinal/duodenal distension. Importantly, no macroscopically identifiable pancreatic tissue was detected in the anatomical region normally located between the stomach, spleen, and duodenum (Fig. 2E).

Together, these findings indicate that limited endocrine/metabolic signals were separable from macroscopically detectable organized pancreatic tissue formation. IPT-treated *Pdx1*-deficient recipients showed detectable circulating insulin in one endpoint cohort and transient glucose increases in a separate survival/blood-glucose cohort, but no macroscopically identifiable organized pancreatic tissue was observed in the examined pancreatic region. Donor-cell localization in *Pdx1*-deficient recipients was not determined in this study; therefore, the KO-arm findings support limited endocrine/metabolic signals rather than anatomical pancreatic tissue formation or sustained physiological correction. To further examine donor-cell heterogeneity and identify markers that may improve future enrichment strategies, we next performed transcriptomic characterization of the E12.5 pancreatic bud.

### Transcriptomic profiling identifies ***Epcam*** as an epithelial enrichment marker associated with the ***Pdx1***^+^ compartment

Because donor-cell composition may influence transplantation outcome, establishing strategies to enrich epithelial fractions containing *Pdx1*-expressing cells is directly relevant to interpreting transplantation results and improving future protocols. Although genetic reporter systems, such as *Pdx1*-GFP transgenic models^23^, enable experimental identification and isolation of pancreatic epithelial populations, a reporter-independent surface marker would provide a more broadly applicable and translationally relevant strategy for prospective enrichment. We therefore reanalyzed published single-cell RNA-sequencing datasets of embryonic mouse pancreas to define *Pdx1*-expressing epithelial populations and prioritize candidate surface markers for prospective enrichment.

**Fig. 3:**
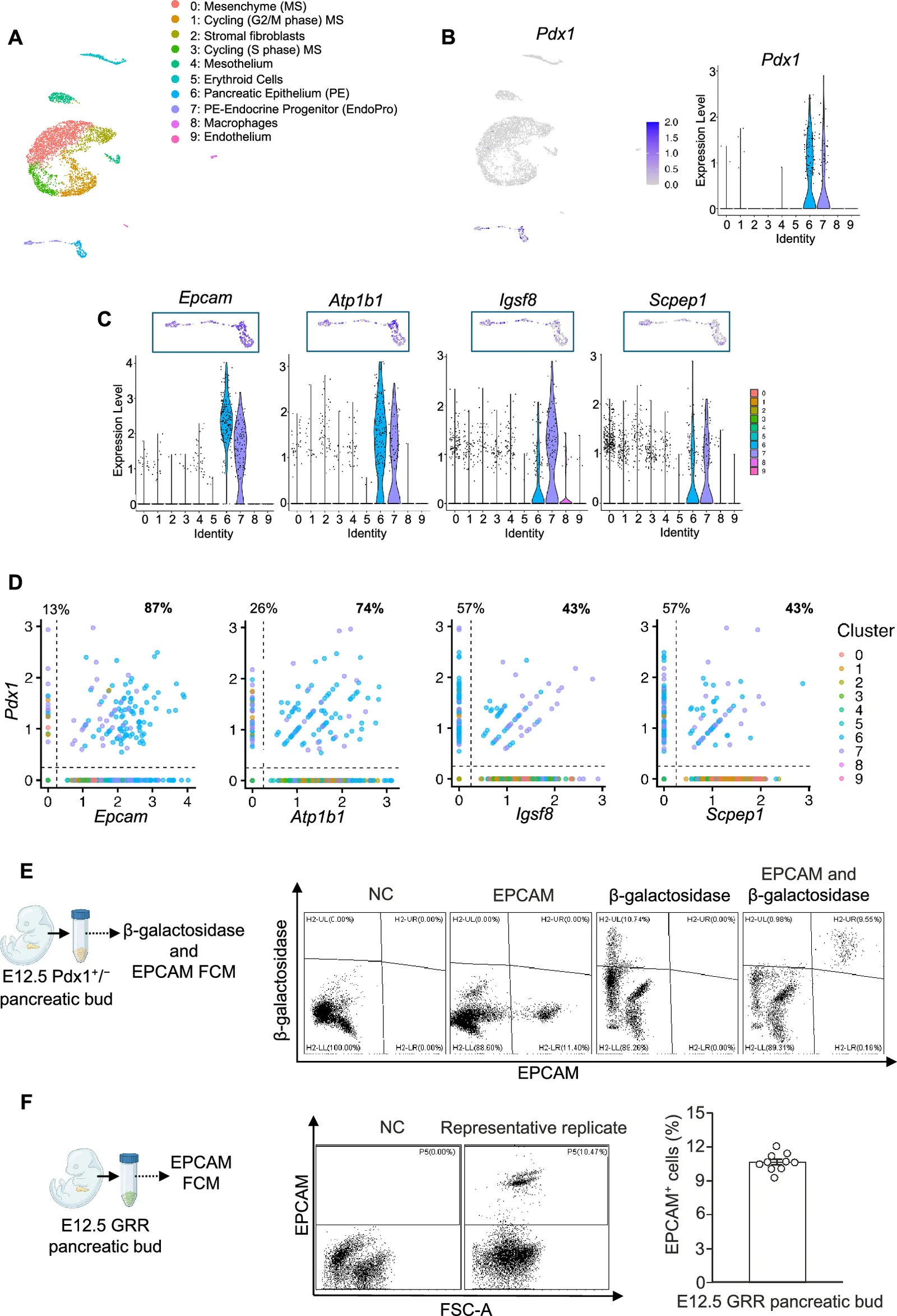
Transcriptomic profiling identifies Epcam as an epithelial enrichment marker associated with the *Pdx1*^+^ compartment. (**A**) UMAP visualization of E12.5 single-cell RNA-sequencing dataset GSM2699156, showing unsupervised clustering of embryonic pancreatic and surrounding cell populations. Ten transcriptionally distinct clusters were identified and annotated based on canonical lineage marker expression. (**B**) UMAP feature plot and violin plot showing *Pdx1* expression across clusters in dataset GSM2699156. *Pdx1* expression is enriched in clusters 6 and 7, corresponding to pancreatic epithelial and epithelial–endocrine progenitor populations. (**C**) Expression distribution of candidate surface markers (*Epcam*, Atp1b1, Igsf8, Scpep1) across clusters. Violin plots show normalized expression levels within each cluster, with inset UMAP feature plots highlighting spatial expression patterns. (**D**) Pseudo-flow-cytometry plots generated from the single-cell RNA-sequencing dataset, showing normalized expression of *Pdx1* versus candidate surface markers (*Epcam*, Atp1b1, Igsf8, Scpep1). Each point represents a single cell colored by cluster identity. Cells above the expression threshold (> 0.25) for each marker were considered marker-positive. The number and percentage of *Pdx1*^+^ cells co-expressing each marker are indicated. (**E**) Experimental validation of EPCAM staining in *Pdx1*^+^ embryonic pancreatic cells. Dissociated pancreatic buds from E12.5 *Pdx1*^LacZ/+^ embryos were analyzed by flow cytometry using Spider-Gal detection of β-galactosidase activity as a reporter of *Pdx1* transcriptional activity together with anti-EPCAM staining. Representative plots show negative control (NC), EPCAM-only staining, β-galactosidase detection, and combined staining demonstrating overlap between EPCAM staining and *Pdx1* reporter activity (one pooled pancreatic bud preparation from n = 5 E12.5 *Pdx1*^LacZ/+^ embryos). (**F**) Independent validation of EPCAM expression in embryonic pancreatic buds from E12.5 GRR (GFP^+^) embryos. Flow cytometric analysis identified a distinct EPCAM^+^ epithelial population within dissociated pancreatic bud cells. Representative plots and quantification of EPCAM^+^ cells are shown. Each point represents an individual embryo (n = 10).

We first analyzed the E12.5 dataset GSM2699156^17^, which contains diverse embryonic pancreatic and surrounding cell populations. Unsupervised clustering resolved 10 transcriptionally distinct clusters, and visualization of the top differentially expressed genes confirmed clear transcriptional separation between clusters (Supplementary Fig. 4). Cluster identities were assigned based on lineage marker expression (Supplementary Fig. 5A, B), and the final annotation is shown in Fig. 3A. Based on the expression of epithelial and pancreatic progenitor markers including *Epcam*, *Cdh1*, *Sox9*, and *Pdx1*, clusters 6 and 7 were annotated as pancreatic epithelial populations. Cluster 6 displayed strong epithelial characteristics, whereas cluster 7 additionally expressed endocrine lineage genes such as *Neurod1*, *Pax6*, and *Chga*, consistent with an epithelial population transitioning toward endocrine progenitors (Fig. 3A, B; Supplementary Fig. 5B).

To identify candidate surface markers associated with *Pdx1*^+^ epithelial cells, genes enriched in the *Pdx1*-expressing epithelial clusters were subsequently intersected with the Cell Surface Protein Atlas^21^, yielding nine candidate surface-associated genes: *Epcam, Atp1b1, F11r, Emb, Tspan13, Ptprf, Tmed4, Igsf8, and Scpep1 (*Fig. 3C*; *Supplementary Fig. 6*).* The top four candidates ranked by average log2 fold change—*Epcam*, *Atp1b1*, *Igsf8*, and *Scpep1*—were selected for downstream visualization and comparative analysis.

To assess how broadly these candidate markers captured the *Pdx1*^+^ compartment, we generated pseudo-flow-cytometry plots from the single-cell RNA-sequencing data, plotting normalized expression of each candidate marker against *Pdx1* expression (Fig. 3D). In GSM2699156, *Epcam* was detected in approximately 87% of *Pdx1*^+^ cells, whereas *Atp1b1*, *Igsf8*, and *Scpep1* were detected in approximately 74%, 43%, and 43% of *Pdx1*^+^ cells, respectively (Fig. 3D). These data identified *Epcam* as the candidate marker capturing the largest fraction of the *Pdx1*-expressing epithelial compartment in this dataset.

Because GSM2699156 contains diverse pancreatic and non-pancreatic cell populations but relatively few epithelial cells, we next analyzed an independent dataset (GSM3140915) derived from E12.5 mouse pancreas enriched for epithelial cells by CD140a-negative selection^17^. Unsupervised clustering resolved 13 transcriptionally distinct populations, and heatmap visualization confirmed clear transcriptional separation between clusters (Supplementary Fig. 7). Cluster annotation based on lineage marker expression is shown in Supplementary Fig. 8A, B and summarized in Supplementary Fig. 9A. In this dataset, clusters 1, 2, 4, 5, and 9 comprised the major *Pdx1*-expressing epithelial populations (Supplementary Fig. 9B). Expression of the candidate markers identified in the first dataset—*Epcam*, *Atp1b1*, *Scpep1*, and *Igsf8*—was confirmed in these populations by UMAP visualization (Supplementary Fig. 9C).

Pseudo-flow-cytometry analysis again revealed that *Epcam* captured the largest proportion of the *Pdx1*^+^ compartment. In this epithelial-enriched dataset, approximately 97% of *Pdx1*^+^ cells expressed *Epcam*, compared with 85%, 45%, and 39% for *Atp1b1, Scpep1*, and *Igsf8*, respectively. Thus, across two independent datasets with distinct cellular compositions, *Epcam* consistently showed the broadest representation across the *Pdx1*-expressing epithelial population. The reciprocal analysis showed that 68% of *Epcam*-positive cells were *Pdx1*-positive, compared with 73%, 58%, and 56% of *Atp1b1*-, *Scpep1*-, and *Igsf8*-positive cells, respectively, under the same thresholding approach (Supplementary Fig. 9D and Supplementary Table 4). Thus, *Epcam* provided the broadest coverage of the *Pdx1*-expressing epithelial population, whereas *Atp1b*1 showed modestly greater enrichment for *Pdx1*^+^ cells but identified a smaller proportion of the total *Pdx1*^+^ compartment. *Scpep1* and *Igsf8* showed both lower coverage of *Pdx1*^+^ cells and a lower proportion of *Pdx1*^+^ cells within their marker-positive populations than *Epcam*. Together, these findings support EPCAM as a practical marker for broad enrichment of the *Pdx1*-expressing epithelial population rather than as a highly specific marker of pancreatic progenitors.

We next performed experimental validation using dissociated E12.5 *Pdx1*^LacZ/+^ pancreatic buds. Flow cytometric analysis combining Spider-Gal detection of β-galactosidase activity with EPCAM staining demonstrated substantial overlap between EPCAM staining and *Pdx1* reporter activity (Fig. 3E; Supplementary Fig. 10A). To confirm that this pattern was not dependent on the reporter allele, we further analyzed pancreatic buds from E12.5 GRR embryos, in which flow cytometry identified a distinct EPCAM^+^ epithelial population (Fig. 3F; Supplementary Fig. 10B). Together, these analyses indicate that EPCAM labels a large fraction of the *Pdx1*-expressing pancreatic epithelial compartment at E12.5, supporting its use as a practical reporter-independent surface marker for enrichment of embryonic pancreatic epithelial populations enriched for *Pdx1*-expressing cells.

## Discussion

In this study, we used intraplacental transplantation (IPT) to compare outcomes after transplantation of dissociated E12.5 pancreatic bud-derived cells in non-KO and *Pdx1*-deficient recipient contexts. The findings support four distinct levels of inference: donor-cell lineage-marker expression, donor-cell detection within pancreatic tissue compartments, formation of an organized pancreatic organ, and sustained correction of host metabolic or survival defects. The non-KO data show that donor-derived cells can be detected in endocrine, acinar, and ductal compartments in the non-KO recipient context. These findings are consistent with the presence of multiple progenitor and differentiating epithelial populations in the E12.5 pancreas, collectively associated with endocrine, ductal, and acinar development^15–18^. Multilineage donor detection in non-KO recipients supports the conclusion that the donor-cell preparation and IPT delivery strategy are compatible with donor-cell detection within pancreatic tissue compartments in the non-KO recipient context.

The R26GRR reporter expresses EGFP from a CAG promoter targeted to the ROSA26 locus, and its original characterization showed widespread expression with tissue-dependent differences in fluorescence intensity^24^. In fibroblast reprogramming, lower CAG promoter activity was associated with smaller cell size and a reprogramming-progressive state^25^. The two GFP-positive clusters in the GRR neonatal-pancreas control differed in both GFP intensity and forward scatter, consistent with pancreatic cell-type or maturation-state heterogeneity accompanied by different CAG-driven reporter activity. Both clusters were included in the total GFP-positive fraction, so this heterogeneity did not affect the donor-cell detection classification.

Despite this non-KO donor-cell detection, transplantation into *Pdx1*^−/−^ recipients did not form macroscopically detectable organized pancreatic tissue. IPT KO recipients showed transient blood-glucose increases in one cohort and detectable serum insulin in a separate endpoint cohort, but no macroscopically identifiable pancreatic structure formed. Because donor-cell localization in *Pdx1*-deficient recipients was not determined, these data do not establish the site, organization, or lineage identity of insulin-producing cells in the KO arm.

This experimental design standardized donor source and IPT protocol across recipient genotypes but did not measure animal-level delivery efficiency, viable cell dose, donor-cell survival during transit, or localization. The comparison therefore shows that organized pancreatic tissue formation was not achieved in *Pdx1*-deficient recipients under the present IPT conditions, while the mechanism remains unresolved. Possible contributors include donor-cell delivery or survival limitations, ectopic localization, developmental timing mismatch, absence or severe disruption of the resident pancreatic epithelial progenitor field, altered epithelial-mesenchymal, vascular, or extracellular-matrix interactions, and broader systemic abnormalities associated with *Pdx1* loss.

Pancreatic organogenesis depends on tightly coordinated epithelial-mesenchymal interactions that regulate progenitor expansion, branching morphogenesis, and lineage specification^6–8^. *Pdx1* itself has also been implicated in pancreatic tubulogenesis and E-cadherin expression^26^. For example, FGF10 signaling from pancreatic mesenchyme maintains proliferation of *Pdx1*-expressing epithelial progenitors and is essential for normal pancreatic bud growth^7,8^. Disruption of these signaling interactions impairs epithelial expansion and branching morphogenesis during pancreas development^7,8^. The failure to detect organized pancreatic tissue in *Pdx1*^−/−^ recipients may reflect absence or severe disruption of the resident pancreatic epithelial progenitor field, altered epithelial-mesenchymal, vascular, or extracellular-matrix interactions, ectopic donor-cell localization, delivery or survival limitations, developmental timing mismatch, or broader systemic defects associated with *Pdx1* loss. The present data do not isolate among these possibilities.

Thus, the KO data should be interpreted as failure to generate macroscopically detectable organized pancreatic tissue under the present IPT conditions, with the limiting mechanism remaining unresolved.

The phenotype of transplanted KO recipients suggests that lethality in the *Pdx1*^−/−^ model is not solely attributable to endocrine insufficiency. *Pdx1* is required for pancreatic development and normal differentiation of the rostral/proximal duodenum, and its loss leads to severe gastrointestinal abnormalities^9^. These findings indicate that assay-specific endocrine/metabolic signals after IPT were insufficient to normalize physiological homeostasis at the cohort level. Grossly examined KO recipients displayed severe growth restriction, dehydration, and duodenal distension, whereas detectable circulating insulin was observed only in a separate endpoint cohort. GFP-positive donor cells were detected in non-KO recipients without exogenous immunosuppression under these fetal transplantation conditions, consistent with the general feasibility of fetal transplantation approaches and fetal immune tolerance settings^27–29^. The present study did not directly assess immune mechanisms.

In parallel with the transplantation experiments, we sought to identify a practical surface marker for enrichment of embryonic pancreatic epithelial fractions containing *Pdx1*-expressing cells. Analysis of two independent single-cell RNA-sequencing datasets revealed that *Epcam* shows the strongest overlap with *Pdx1*-expressing epithelial cells in the E12.5 mouse pancreatic bud. The less-than-complete overlap observed in scRNA-seq likely reflects, in part, the technical sparsity of single-cell transcriptomic data, in which expressed transcripts may be missed because of dropout and limited capture efficiency^30–32^. Together with our flow-cytometric validation, these findings support EPCAM as a practical reporter-independent surface marker for enriching a large fraction of the embryonic mouse *Pdx1*^+^ epithelial compartment.

Prior literature supports EPCAM (epithelial cell adhesion molecule) primarily as a transmembrane glycoprotein mediating calcium-independent epithelial cell adhesion rather than as a pancreas-specific regulator^33–35^. In the pancreatic context, functional studies in the human fetal pancreas showed that EPCAM mediates epithelial cell–cell adhesion and is developmentally regulated during islet morphogenesis^36^, whereas our mouse embryonic transcriptomic analyses support association of *Epcam* with *Pdx1*-expressing epithelial compartments. However, the present study evaluates EPCAM as a practical enrichment marker in the E12.5 mouse pancreas and does not establish that EPCAM itself functionally regulates progenitor maintenance, morphogenesis, or transplantation outcome. The reciprocal pseudo-flow analysis also showed that the EPCAM-positive fraction was not exclusively *Pdx1*-positive (68% *Pdx1*-positive; Supplementary Table 4), reinforcing that EPCAM should be interpreted as an epithelial enrichment marker rather than a specific pancreatic progenitor marker.

Previous studies have associated several epithelial markers with embryonic pancreatic epithelial populations. Developmental epithelial fractionation analyses have associated EPCAM and CDH1 with the pancreatic epithelial compartment in mouse embryonic pancreas^19^. Likewise, *Cdh1* and *Cldn6* have been used to annotate epithelial clusters in single-cell RNA-sequencing datasets of the developing pancreas^20^. Because of these previous reports, we also evaluated *Cdh1* and *Cldn6* in our analysis. UMAP visualization confirmed that both genes are enriched within the pancreatic epithelial compartment (Supplementary Fig. 10C). However, pseudo-flow-cytometry analysis revealed important differences in their overlap with the *Pdx1*^+^ population. Approximately 55% of *Pdx1*^+^ cells expressed *Cdh1*, indicating that *Cdh1* alone would recover only a subset of the *Pdx1*-expressing epithelial compartment (Supplementary Fig. 10D). In contrast, *Cldn6* showed high transcriptomic overlap (~ 95%) with *Pdx1*^+^ cells.

Although *Cldn6* showed high transcriptomic overlap, its suitability for prospective live-cell isolation would require validation of an antibody recognizing an extracellular epitope under non-permeabilizing conditions. In contrast, EPCAM is a well-established extracellular surface marker that is readily accessible to antibodies and has been widely used for epithelial-cell isolation^37^. Mouse live-cell epithelial isolation workflows also support the feasibility of prospective epithelial-cell recovery by flow-cytometric sorting^38–40^. Thus, although both *Epcam* and *Cldn6* show strong transcriptomic representation within pancreatic epithelial cells, *Epcam* provides a more practical marker for enrichment of viable pancreatic epithelial cells within preparations containing *Pdx1*-expressing cells.

These developmental findings also have implications for pancreatic cell-replacement strategies. Many current pancreatic cell-replacement approaches emphasize optimization of donor-cell differentiation state and functional maturity^41^. However, our results show that the transplanted E12.5 pancreatic bud-derived donor population did not form macroscopically detectable organized pancreatic tissue in *Pdx1*-deficient recipients under the present IPT conditions. This observation underscores the importance of evaluating donor-cell behavior, delivery route, timing, and recipient developmental context when interpreting tissue-formation outcomes. More complete tissue formation may require not only appropriately specified donor cells but also a recipient developmental context capable of supporting their survival, morphogenesis, spatial localization, and maturation. In blastocyst complementation models, donor pluripotent stem cells are introduced at a much earlier developmental stage and can participate more broadly in the host developmental program, potentially contributing across multiple tissue compartments during organ formation^42–44^. In contrast, IPT delivers dissociated pancreatic bud-derived cells after early organ-field patterning has begun^6^, when recipient mesenchymal and vascular environments are already developing. The present IPT results therefore do not directly test blastocyst-complementation mechanisms; rather, they show that this later systemic transplantation strategy did not form organized pancreatic tissue in *Pdx1*-deficient recipients.

Compared with the previous human iPSC-derived PDX1^+^ progenitor IPT study^12^, the present mouse pancreatic bud donor experiment produced a similar qualitative KO-arm outcome: limited endocrine/metabolic signals without macroscopically detectable organized pancreatic tissue formation. The current study additionally shows that E12.5 pancreatic bud-derived cells can be detected within endocrine, acinar, and ductal compartments in a subset of non-KO recipients. These non-KO findings provide a donor-cell detection and compartmental-localization context for interpreting the KO arm, but they do not identify the limiting mechanism in *Pdx1*-deficient recipients.

Several limitations of this study should be noted. First, the present transplantation experiments assess donor-cell detection at the tissue level using GFP tracing and immunohistochemistry, but do not include quantitative morphometric analysis of recipient pancreatic tissue. Therefore, descriptions of multilineage donor detection are based on qualitative histological observation and should be interpreted accordingly. We did not assess mature β-cell identity using markers such as MAFA or NKX6.1, nor did we measure circulating C-peptide or regulated insulin secretion. Therefore, GFP/insulin overlap should be interpreted as endocrine-marker expression rather than evidence of mature β-like cell function. Second, the sample sizes for KO recipient analyses are relatively small, reflecting the inherent constraints of the KO model in which affected animals die within the first postnatal week. While the assay-specific survival, glucose, and insulin observations are consistent with limited endocrine/metabolic signals after IPT, larger cohorts would be needed to determine the reproducibility and magnitude of these observations with greater statistical confidence. Third, the IPT delivery method results in systemic distribution of donor cells. The factors determining donor-cell-detected versus non-detected endpoint samples, including injection efficiency, vascular access, donor-cell survival during transit, and access to appropriate mesenchymal, vascular, and extracellular-matrix support, remain incompletely characterized. Available endpoint records were not structured to test correlations between donor pool, litter or experiment date, recipient genotype, endpoint donor-cell detection, and glucose or insulin outcomes; these sources of heterogeneity therefore remain unresolved. Fourth, the *Pdx1*^−/−^ model involves systemic developmental defects beyond pancreatic agenesis, such as duodenal abnormalities^9^. It is therefore not possible to attribute the lack of organized pancreatic tissue formation solely to the local pancreatic developmental context; systemic physiological compromise may also contribute to the inability of transplanted cells to form organized tissue. In addition, gross morphology was documented qualitatively rather than as a prespecified quantitative scoring dataset; body weight, body-size measurements, hydration score, subcutaneous adipose-tissue status, and gastrointestinal or duodenal distension were not collected systematically for group-wise morphometric analysis. Fifth, although our comparative design standardized donor source, donor preparation, and intraplacental transplantation conditions while varying recipient genotype, we cannot exclude the possibility that donor stage also influences tissue-formation outcome. In the present study, dissociated E12.5 pancreatic bud cells were detected within pancreatic tissue in non-KO recipients but did not form macroscopically detectable organized pancreatic tissue in *Pdx1*^−/−^ recipients. Direct donor-cell localization in *Pdx1*-deficient recipients was not determined because the available KO endpoint material was allocated to survival/blood-glucose monitoring or serum insulin measurement rather than localization-preserved tissue analysis. Therefore, the current data do not distinguish whether the KO outcome reflects donor-cell localization, delivery efficiency, founder-cell limitation, donor-cell survival, developmental timing, absence or severe disruption of the resident pancreatic epithelial progenitor field, or broader systemic abnormalities caused by *Pdx1* deficiency. Directly testing earlier embryonic donor stages in this system is technically challenging because donor tissue yield is substantially lower, and prospective enrichment strategies such as EPCAM-based isolation would be expected to further reduce recoverable cell number, making transplantation at the required scale difficult. Finally, we cannot exclude the possibility that dissociation of the E12.5 pancreatic bud disrupts cell–cell interactions or local tissue-derived signals that are important for maintaining tissue-forming potential. Future studies using intact tissue or minimally dissociated organoid approaches may help address this question.

In summary, this study shows that E12.5 pancreatic bud-derived donor cells can be detected within endocrine, acinar, and ductal compartments in the pancreata of non-KO recipients retaining an endogenous pancreatic developmental field. However, transplantation into *Pdx1*-deficient embryos did not generate macroscopically detectable organized pancreatic tissue; subsets showed detectable circulating insulin or transient glucose increases in assay-specific cohorts, but donor-cell localization and mechanism remain unresolved. Under the present IPT conditions, the experiments support donor-cell detection within pancreatic compartments in a subset of non-KO recipients, but not formation of an organized pancreas or sustained physiological correction in *Pdx1*-deficient recipients. Separately, our transcriptomic and experimental analyses identify EPCAM as a practical reporter-independent surface marker that marks a large fraction of the *Pdx1*-expressing epithelial compartment in the E12.5 pancreatic bud. Together, these results highlight the need to evaluate donor-cell state together with delivery route, localization, and recipient developmental context in future transplantation studies.

## Methods

### Animals

*Pdx1* mutant mice carrying a LacZ insertion in one *Pdx1* allele were used as previously described^9,12^. To generate experimental litters, *Pdx1*^+/−^ mice were maintained on a BDF1 background by crossing with wild-type B6D2F1 mice. Offspring included *Pdx1* knockout (KO) mice and non-knockout littermates, the latter comprising *Pdx1*^+/+^ and *Pdx1*^+/−^ animals. *Emb*ryonic day (E) was defined based on the presence of a vaginal plug, designated as E0.5, and postnatal day (P) was defined as the day of birth (P0). C57BL/6N-Gt(ROSA)26Sor/Rbrc mice were used as a source of green fluorescent protein–positive (GFP^+^) donor cells and tissues^24^. All mice were housed under specific pathogen-free conditions at the University of Tsukuba Laboratory Animal Resource Center with ad libitum access to food and water. All animal experiments were approved by the University of Tsukuba Animal Ethics Committee (approval no. 23-039) and performed in accordance with institutional and national guidelines and the ARRIVE guidelines^45^. *Emb*ryonic and neonatal mice of both sexes were used. Sex was not determined for embryonic-stage analyses.

### Allocation and inclusion/exclusion criteria

Because recipient genotype was unknown at the time of intraplacental transplantation, all embryos in injected litters underwent IPT, and genotyping was performed only after birth. Among live-born recipients, all KO mice (n = 22) were allocated to KO outcome analyses and assigned to assay-specific endpoint cohorts: survival/blood-glucose monitoring (n = 15) or early serum insulin measurement by ELISA (n = 7). These endpoint cohorts were separate, so serum insulin values were not correlated with survival/blood-glucose trajectories in the same animals. Of the 15 IPT KO animals assigned to survival/blood-glucose monitoring, 13 contributed blood-glucose trajectories; pups that died on P0 were included in survival analysis but excluded from the blood-glucose panel. Of the 64 live-born non-KO recipients, 32 were used for downstream analyses according to assay allocation and sample availability: recipient-pancreas flow cytometry (n = 17, including 10 mice also used for fluorescence stereomicroscopy), immunohistochemistry (n = 8), and a non-KO blood-glucose reference cohort (n = 7). The remaining 32 non-KO recipients were not included in endpoint analyses. Selection of non-KO recipients for these assays was made after birth based on practical assay allocation and not on transplantation outcome. Inclusion and exclusion criteria were defined before analysis. For non-KO recipients, flow-cytometry endpoint samples were classified as donor-cell detected when GFP-positive cells were detected above background.

### Euthanasia

Mice were euthanized using age-appropriate methods. Adult and pregnant mice were euthanized by CO_2_ inhalation. Neonatal mice (P0–P4) were euthanized by hypothermia-induced anesthesia on ice followed by decapitation.

### Genotyping

Genotypes were determined by PCR analysis of tail DNA using the following primers for the *Pdx1* locus: *Pdx1*_FW, 5′-ATTGAGATGAGAACCGGCATG-3′; *Pdx1*-*LacZ*_FW, 5′-TGTGAGCGAGTAACAACC-3′; and *Pdx1*_RV, 5′-TTCATGCGACGGTTTTGGAAC-3′.

For transplantation experiments, recipient embryos were obtained from timed matings of *Pdx1*^+/−^ × *Pdx1*^+/−^ mice. The morning on which a vaginal plug was detected was designated E0.5.

### β-galactosidase (LacZ) staining

For whole-mount tissue staining, one pregnant *Pdx1*^+/−^ dam was euthanized at E12.5, and 7 embryos were harvested in ice-cold phosphate-buffered saline (PBS) under a stereomicroscope. Tail samples were collected from each embryo for subsequent PCR genotyping. To identify the pancreatic primordia, the stomach and associated mesenteric structures were exposed according to established embryological landmarks^17^. The dorsal pancreatic bud was identified within the dorsal mesogastrium between the stomach and the developing splenic primordium, whereas the ventral pancreatic bud was identified near the stomach–duodenum junction. The stomach, including the pancreatic buds and adjacent duodenum, was microdissected and processed using a β-galactosidase staining kit (Cell Biolabs, Cat. No. AKR-100) according to the manufacturer’s instructions. This whole-mount preparation was used to validate anatomical landmarks; donor-cell preparation for IPT used microdissected pancreatic bud tissue as described below.

For dissociated-cell β-galactosidase staining, pancreatic bud tissue was collected from a separate E12.5 *Pdx1*^+/−^ pregnant dam yielding 8 embryos in total. Tail samples were collected from each embryo for subsequent PCR genotyping. In parallel, pancreatic bud regions were carefully microdissected using fine forceps with minimal contamination from surrounding mesenchymal tissue. Tissues were transferred into 1.5-mL microcentrifuge tubes containing PBS and mechanically dissociated into single-cell suspensions by repeated passage through a 25-gauge needle attached to a 1-mL syringe. Cell suspensions were filtered through a 35-μm cell strainer snap cap (Falcon, Cat. No. 352235) to remove aggregates and were then stained using the same β-galactosidase staining kit according to the manufacturer’s instructions.

Wild-type embryos processed in parallel served as negative controls. Stained tissues and dissociated cells were imaged by bright-field microscopy. PCR genotyping was performed after staining, and the staining patterns were confirmed to be concordant with the genotyping results. Representative images are shown in Supplementary Fig. 1.

### Donor cell preparation

E12.5 GRR (GFP^+^) embryos were harvested in ice-cold phosphate-buffered saline (PBS). Pancreatic buds were microdissected under a stereomicroscope and mechanically dissociated to obtain a single-cell suspension. The transplanted inoculum was not purified or EPCAM-sorted and should be interpreted as a mixed E12.5 pancreatic bud-derived preparation containing pancreatic epithelial and associated non-epithelial cells. Cells were pooled in 1 tube and centrifuged at 300 × g for 5 min, washed once with PBS, and resuspended in ice-cold PBS at a final concentration of 1 × 10^4^ cells μL^−1^ for intraplacental transplantation (IPT). On average, approximately 4 × 10^4^ pancreatic bud cells were obtained per E12.5 embryo. Cell viability was not formally assessed prior to transplantation in this study. However, the mechanical dissociation protocol was performed rapidly under ice-cold conditions to minimize cell damage, and donor-cell detection in non-KO recipients is consistent with viability of at least some transplanted cells.

### Intraplacental transplantation (IPT)

IPT was performed as previously described^12–14^. Briefly, timed-pregnant *Pdx1*^+/−^ mice carrying E9.5 embryos were anesthetized with isoflurane and maintained on a temperature-controlled heating pad at 37 °C throughout the procedure. After a midline laparotomy, the uterine horns were gently exteriorized under a stereomicroscope. Donor-cell suspensions were loaded into pulled glass microcapillaries using flexible aspiration tubing (Drummond, Cat. No. 68-0449-50) and connected to a microinjector system (FemtoJet; Eppendorf). Each placenta was carefully punctured, and approximately 1 μL of donor-cell suspension (1 × 10^4^ cells μL^−1^, corresponding to ~ 1 × 10^4^ cells per embryo) was injected into the placental labyrinth at a controlled pressure of 60–70 hPa. Following injection, the uterus was returned to the abdominal cavity, the abdominal wall was sutured, and the dam was maintained at body temperature until recovery from anesthesia. Across all experiments, the live birth rate after IPT was recorded. Offspring were genotyped postnatally to classify recipients as non-KO or KO.

### Fluorescence stereomicroscopy

Mice were euthanized by decapitation at P1, and their pancreata were dissected for immediate imaging. A fluorescence stereomicroscope (MZFLIII; Leica, Nussloch, Germany) was used for ex vivo analysis of green fluorescent protein (GFP).

### Immunohistochemistry (IHC)

Pancreatic tissue samples from 8 transplanted non-KO mice (IPT non-KO), 1 non-transplanted wild-type mouse (GFP^−^ control), and 1 non-transplanted GRR mouse (GFP^+^ control), all collected at 2 months of age, were fixed in Mildform 10N (FUJIFILM Wako, Cat. No. 133-10311) and embedded in paraffin. Paraffin blocks were sectioned at 7 μm using a Sliding Microtome SM2000R (Leica Biosystems, Wetzlar, Germany). Sections were subjected to antigen retrieval in antigen retrieval buffer (Agilent Technologies, Cat. No. S1699) for 10 min at 110 °C. After blocking with 5% goat serum and 1% bovine serum albumin in PBS, sections were incubated with primary antibodies overnight at 4 °C. Primary antibodies included chicken anti-GFP (Aves, Cat. No. GFP-1010, 1:500), rabbit anti-glucagon (Cell Signaling Technology, Cat. No. 2760S, 1:1000), and guinea pig anti-insulin (Thermo Fisher Scientific, Cat. No. PA1-26938, 1:100). For no-primary-antibody control sections, blocking solution was applied in place of the primary antibodies. Sections were then washed three times in PBST for 10 min each. Secondary antibodies, including goat anti-chicken Alexa Fluor 488 (Invitrogen, Cat. No. A11039, 1:200), goat anti-guinea pig Alexa Fluor 594 (Invitrogen, Cat. No. A11076, 1:500), and donkey anti-rabbit Alexa Fluor 350 (Invitrogen, Cat. No. A10039, 1:500), were applied for 1 h at room temperature. Sections were washed three times in PBST for 10 min each. For GFP-only staining, sections were mounted with VECTASHIELD Vibrance with DAPI (Vector Laboratories, Cat. No. H-1800). For GFP–insulin–glucagon staining, sections were mounted with DPX neutral mounting medium (Sigma, Cat. No. 317616). Images were acquired using a BIOREVO BZ-X800 microscope (Keyence, Osaka, Japan). All transplanted non-KO recipients (n = 8) were processed for staining. Donor-derived GFP-positive pancreatic regions were detected in 5 of 8 transplanted mice, and only these animals were included in quantitative analyses and representative images (Supplementary Fig. 3; Supplementary Table 2). For Fig. 1G–I, endocrine-marker overlap was quantified using custom Python scripts with NumPy, pandas, Pillow, SciPy ndimage, and Matplotlib. Single-channel 8-bit GFP, insulin, glucagon, and merged image exports were cropped to a shared field size and background-corrected by Gaussian filtering. Islet ROIs were defined from provisional GFP/insulin masks with manual ROI masks where available; GFP positivity used a fixed threshold derived from GFP-negative controls, and insulin and glucagon masks were defined using ROI-based Otsu thresholds^46^. Endocrine area was defined as insulin-positive or glucagon-positive area within the ROI, and donor endocrine-marker overlap was defined as GFP-positive area overlapping endocrine-marker-positive area. Quantification for Fig. 1G–I was restricted to the three transplanted non-KO samples with GFP-positive endocrine-islet signal; no-IPT values were used only as background/reference measurements.

### Flow cytometry (FCM) analysis

For evaluation of GFP-positive donor-cell detection, neonatal recipient pancreata (P0–P3) were dissociated in Collagenase Type IV (Gibco, Cat. No. 17104019) for 15 min at 37 °C, collected in PBS containing 10% FBS, filtered through 40-μm cell strainers, and centrifuged at 300 × g for 5 min. Cell pellets were resuspended in FACS buffer (1% FBS in PBS) and incubated with PE-conjugated anti-mouse CD45 (BioLegend, Cat. No. 103105) for 20 min on ice. After staining, cells were washed twice with FACS buffer and resuspended in 200 μL FACS buffer containing DAPI (Dojindo, Cat. No. 340-07971) for viability assessment. Data acquisition and analysis were performed using a CytoFLEX flow cytometer (Beckman Coulter), and 20,000 events were initially recorded per sample. For gating and compensation controls, pancreatic cell suspensions from 4 wild-type neonatal mice were used for unstained, all-stained, CD45-only, and DAPI-only control tubes, and pancreatic cell suspensions from 2 GRR neonatal mice were used for unstained and all-stained control tubes.

For evaluation of EPCAM and *Pdx1* reporter activity in embryonic pancreatic buds, pancreatic buds from 5 E12.5 *Pdx1*^+/−^ embryos were pooled and dissociated in 0.5 mL TrypLE in a 1.5-mL tube. After mechanical dissociation, samples were incubated at 37 °C for 10 min with pipetting at 5-min intervals. PBS containing 10% FBS (1 mL) was then added, and the suspension was filtered through a 35-μm cell strainer snap cap (Falcon, Cat. No. 352235) to remove aggregates. To detect *Pdx1* (*LacZ*) activity, cells were loaded with Spider-Gal (Dojindo, Cat. No. SG02) according to the manufacturer’s instructions and incubated for 20 min at 37 °C. Cells were then washed and stained with APC-conjugated anti-EPCAM (Invitrogen, Cat. No. 17-5791-82) and PE-Cy7-conjugated anti-CD45 (BioLegend, Cat. No. 103113) for 20 min at 4 °C. After two washes with FACS buffer, cells were resuspended in 200 μL FACS buffer containing DAPI for viability assessment. Data acquisition and analysis were performed using a CytoFLEX flow cytometer, and 20,000 events were initially recorded. For this experiment, the pooled *Pdx1*^+/−^ bud sample was divided into 2 tubes: one fully stained test sample and one Spider-Gal-only control. In parallel, pancreatic buds from 6 wild-type E12.5 embryos were pooled and divided into 4 control tubes: unstained, DAPI-only, EPCAM-only, and CD45-only. Flow-cytometry antibodies were used at the manufacturer-recommended amounts per test, according to the corresponding product datasheets.

For evaluation of EPCAM expression in GRR (GFP^+^) embryonic buds, ten E12.5 GRR embryos were analyzed as individual fully stained samples for quantification. Additional GRR bud material was used for control tubes: unstained, DAPI-only, CD45-PE-only, and EPCAM-APC-only. After staining and washing, cells were resuspended in a FACS buffer containing DAPI for viability assessment. Data acquisition and analysis were performed using a CytoFLEX flow cytometer, and 20,000 events were initially recorded per sample.

### Physiological and survival analysis of KO recipients

#### Survival analysis

Newborn pups were individually identified by limb tattooing on the day of birth. Genotyping was performed using tail-tip samples. To support maternal care, dams were provided with CMF sprouts (Oriental Yeast, Tokyo, Japan). Survival of newborn mice was monitored daily until all *Pdx1*^−/−^ recipients had died.

#### Blood glucose measurement

A small volume of blood was collected daily from P0 until the day before death by facial vein puncture. Blood glucose levels were measured using a glucometer (Terumo, Tokyo, Japan). The lower detection limit of the assay was 20 mg dL^−1^; values below this limit were recorded as 20 mg dL^−1^ for analysis. Individual blood-glucose measurements are provided in Supplementary Table 3.

#### Serum insulin measurement

Serum samples were collected from P1–P4 mice after hypothermia-induced anesthesia on ice followed by decapitation. Blood was collected after decapitation, kept on ice for 15 min to clot, and centrifuged at 2000 × g for 20 min at 4 °C. Serum was collected, stored at −80 °C, and later used for ELISA. Sampling was performed across P1–P4 because *Pdx1*-deficient neonates deteriorated and died within a narrow early postnatal window, and serum was collected at available endpoint times rather than at a prospectively fixed age; therefore, these values were interpreted as endpoint detectability readouts rather than age-controlled insulin-level comparisons. Insulin concentrations were measured using an Ultrasensitive Mouse Insulin ELISA kit (Moringa, Cat. No. M1104) according to the manufacturer’s instructions. Optical density was measured at 450 nm using a SpectraMax M5 microplate reader (Molecular Devices) within 30 min of assay completion.

#### Morphological assessment

Available gross morphological records from KO and non-KO neonates were reviewed qualitatively, including body size, hydration status, subcutaneous adipose tissue, and gastrointestinal/duodenal distension. The abdominal cavity was examined for the presence or absence of pancreatic tissue when anatomical records were available. The number of animals with complete gross morphological records was not prospectively predefined; therefore, these observations are reported as qualitative records only.

### Data visualization and statistical analysis

Data visualization and statistical analyses were performed using SVG Plotter v10 (Hamada, 2026; available at https://miiichiii.github.io/figure-making/). Survival analysis was performed using the log-rank (Mantel-Cox) test, as indicated in the Fig. 2 legend. For Fig. 1D and Fig. 2C, endpoint detection data are presented descriptively as individual values and detection frequencies because the displayed categories were defined by the same detection readout used for plotting. *P* < 0.05 was considered statistically significant for prespecified statistical comparisons. No formal randomization or blinding procedures were used.

### Experimental unit

Experimental units differed by assay. For transplantation, survival, blood glucose, serum insulin, fluorescence imaging, and recipient-pancreas flow-cytometry analyses, the experimental unit was one mouse. For embryonic whole-mount and dissociated-cell β-galactosidase staining in Supplementary Fig. 1, the experimental unit was one embryo. For EPCAM/β-galactosidase flow-cytometry analysis using pooled *Pdx1*^LacZ/+^ pancreatic buds, the experimental unit was one pooled bud preparation. For EPCAM flow-cytometry analysis of E12.5 GRR pancreatic buds, the experimental unit was one embryo. No formal a priori sample size calculation was performed. Sample sizes were determined by the availability of timed pregnancies, the technical constraints of intraplacental transplantation, and the limited postnatal survival of *Pdx1*-deficient mice. The study was conducted as an exploratory proof-of-concept analysis.

### Bioinformatics and surface marker confirmation

Publicly available single-cell RNA-sequencing datasets of E12.5 embryonic mouse pancreas (GSM2699156 and GSM3140915) were analyzed using Seurat v5.4.0^47^ in R v4.5.2 to identify *Pdx1*-expressing epithelial populations and prioritize candidate surface markers for prospective enrichment. Raw count matrices were imported with ReadMtx, and Seurat objects were generated using CreateSeuratObject. Cells with < 200 or > 8000 detected genes, or > 5% mitochondrial transcripts, were excluded. Mitochondrial gene content was calculated using PercentageFeatureSet with the pattern ^mt-. Data were normalized by LogNormalize (scale factor = 10,000), and the top 2000 variable genes were identified using the vst method. Scaled data were subjected to principal component analysis, and graph-based clustering was performed using the first 10 principal components at a resolution of 0.2. Clusters were visualized by Uniform Manifold Approximation and Projection (UMAP)^48^. Differentially expressed genes for each cluster were identified using FindAllMarkers with the Wilcoxon rank-sum test, and the top marker genes per cluster were visualized by heatmap to evaluate transcriptional separation between clusters.

Cell-type annotation was performed independently for each dataset using a two-step strategy combining unbiased marker discovery with curated lineage validation. Cluster identities were assigned based on expression of canonical markers representing major pancreatic developmental compartments, including mesenchymal/stromal cells (*Col1a1, Col1a2, Col3a1, Lum, Dcn, Pdgfra, Pdgfrb, Rgs5*), mesothelium (*Wt1, Pdpn, Upk3b*), epithelium (*Epcam, Cdh1, Krt8, Krt18, Sox9*), pancreatic progenitors (*Pdx1, Nkx6-1, Ptf1a*), endocrine lineage cells (*Neurog3, Neurod1, Pax6, Chga, Ins1, Gcg, Sst*), endothelium (*Pecam1, Cdh5, Kdr, Emcn*), immune/macrophage populations (*Lyz2, Tyrobp, C1qa, Adgre1, Fcgr1*), erythroid cells (*Hba-a1, Hbb-bt, Alas2*), and cycling populations (*Mki67, Top2a, Ccnb1, Tyms*). For datasets showing greater stromal or epithelial heterogeneity, additional focused marker panels were used to refine annotation. For improved visualization of *Pdx1* expression in the UMAP, the maximum color scale was set to 2.

To identify candidate surface markers associated with *Pdx1*-expressing epithelial populations, clusters annotated as pancreatic epithelium or epithelial/endocrine progenitor populations were selected for downstream analysis. Differentially expressed genes in these clusters were identified using Seurat’s FindAllMarkers function. Candidate genes were filtered using an average log2 fold change > 1 and an adjusted *P* value < 0.001. Only genes meeting both criteria were retained for further analysis.

The resulting gene list was intersected with the Cell Surface Protein Atlas^21^ to prioritize proteins with annotated cell-surface localization suitable for prospective cell isolation. Expression patterns of shortlisted candidates were then examined by UMAP visualization and violin-plot analysis across annotated clusters.

To estimate how broadly each candidate marker captured the *Pdx1*-expressing compartment, normalized single-cell expression values were visualized as pseudo-flow-cytometry scatter plots, with *Pdx1* expression on one axis and candidate-marker expression on the other. Cells were classified as positive or negative for each gene based on predefined expression thresholds. The proportion of *Pdx1*-positive cells co-expressing each candidate marker was calculated as the fraction of double-positive cells among all *Pdx1*-positive cells (i.e., marker^+^*Pdx1*^+^ / total *Pdx1*^+^ cells). Expression thresholds for defining marker-positive cells were set at > 0.25 (normalized expression) for GSM2699156 and > 0.15 for GSM3140915, based on the distribution of expression values in each dataset. The lower threshold for GSM3140915 reflects the epithelial enrichment of this dataset, which shifts overall expression distributions. The relative ranking of candidate markers was consistent across both datasets despite the different thresholds, supporting the robustness of the analysis. For the reciprocal EPCAM analysis, the proportion of *Epcam*-positive cells co-expressing *Pdx1* was calculated as double-positive cells divided by all *Epcam*-positive cells (Supplementary Table 4).

## Supplementary Information

Supplementary Information 1.

Supplementary Information 2.

Supplementary Information 3.

Supplementary Information 4.

Supplementary Information 5.

## Data Availability

This study did not generate new sequencing data. Publicly available datasets were re-analyzed from GEO under accession GSE101099, including samples GSM2699156 and GSM3140915. Other data supporting the findings of this study are available from the corresponding author upon reasonable request.

## Use of generative artificial intelligence

ChatGPT (OpenAI) was used during manuscript preparation to assist with language polishing, translation, improvement ofsentence and text structure, and drafting and refinement of explanatory text. All AI-assisted content and suggestions werecritically reviewed and verified by the authors against the underlying data and relevant literature. Scientific interpretation,judgment, and final conclusions remained the responsibility of the authors, who take full responsibility for the accuracy andintegrity of the manuscript.
