# Supplementary material for: E12.5 pancreatic bud-derived cells are detected in recipient pancreata after intraplacental transplantation, but Pdx1-deficient recipients lack a macroscopically detectable pancreas
Source: Sci Rep. 2026 Sep 14;16:28207. doi: 10.1038/s41598-026-65314-w (PMC13575222; doi:10.1038/s41598-026-65314-w)
Supplement: Supplementary file 1 [file 41598_2026_65314_MOESM1_ESM.pdf]

Supplementary Figure 1

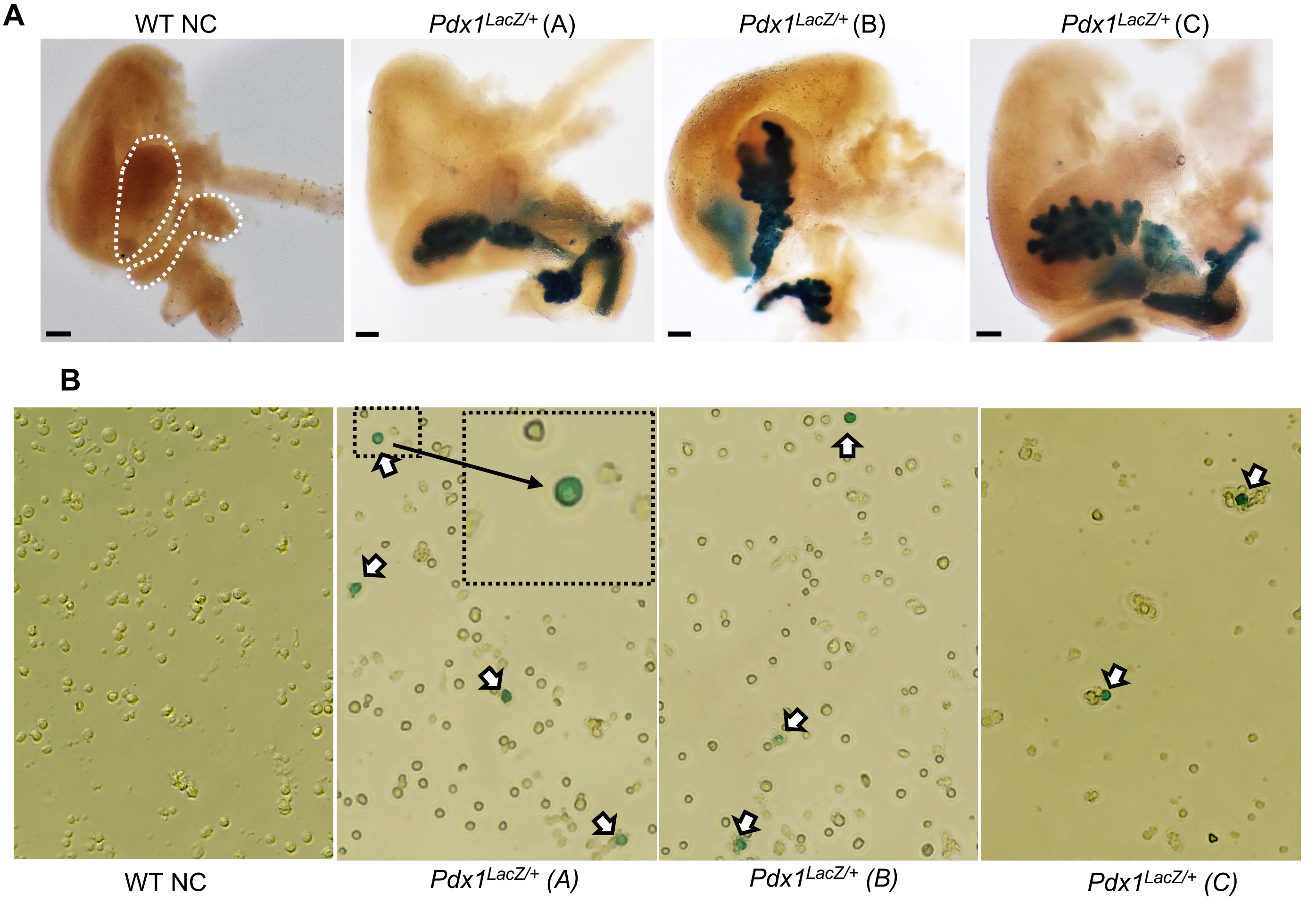

Supplementary Figure 1 | Validation of embryonic pancreatic bud-region isolation using *Pdx1* reporter activity. (A) Whole-mount β-galactosidase staining of anatomically matched E12.5 stomach-duodenum-pancreatic bud regions from wild-type negative control (WT NC) and *Pdx1*$^{LacZ/+}$ (*Pdx1*$^{+/-}$) embryos. The dashed outline indicates the pancreatic bud-region collected for donor-cell preparation. No staining was detected in WT NC tissue, whereas strong reporter activity was detected in *Pdx1*$^{LacZ/+}$ embryos. Scale bar, 0.1 mm. (B) β-galactosidase staining of dissociated single-cell suspensions derived from microdissected tissues. Robust blue staining was detected in samples obtained from *Pdx1*$^{LacZ/+}$ embryos, whereas anatomically matched cells from wild-type control tissue showed no detectable signal, confirming accurate isolation of Pdx1^{+} pancreatic progenitor cells.

Supplementary Figure 2

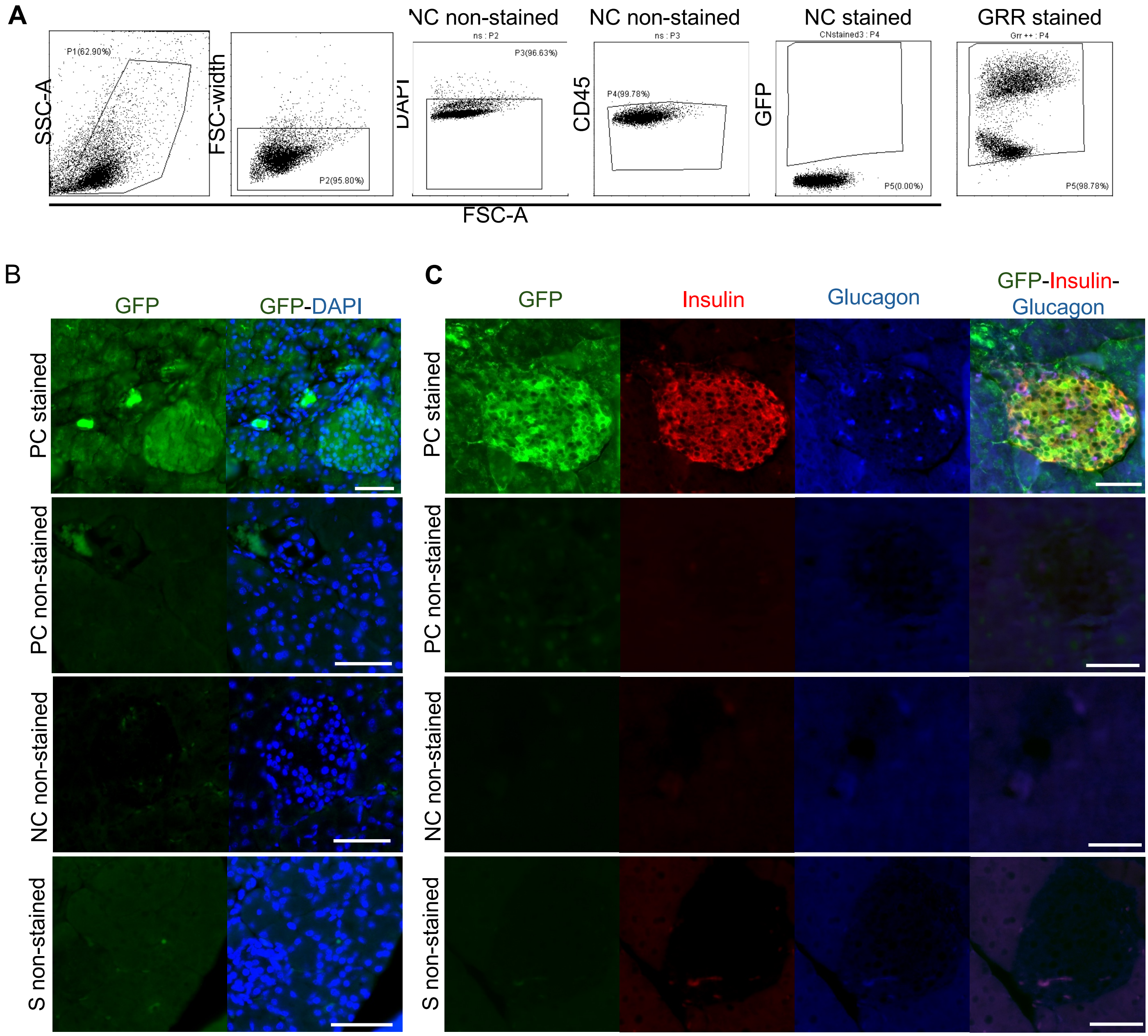

Supplementary Figure 2 | Validation of immunostaining specificity and flow cytometric gating strategy

(A) Flow cytometry gating strategy used for quantification of donor-derived cells. Sequential gates were applied to exclude debris (FSC/SSC), doublets (FSC-width vs FSC-area), dead cells (DAPI$^+$), and CD45$^+$ hematopoietic cells prior to GFP analysis. The all-stained GRR neonatal-pancreas positive control shows two GFP-positive clusters differing in GFP intensity and forward scatter; both were included in the total GFP-positive fraction and were not phenotyped separately. (B) Immunofluorescence controls for GFP detection. Positive control (PC) pancreatic sections derived from GFP$^+$ tissue show robust GFP signal, whereas no-primary antibody controls in sample (S non-stained) and non-transplanted negative control (NC) tissues exhibit no detectable fluorescence, confirming staining specificity. Nuclei were counterstained with DAPI. Scale bars = 50 μm. (C) Validation of endocrine marker staining. Immunostaining for GFP, insulin, and glucagon demonstrates expected signal detection in positive control sections, while omission of primary antibodies abolishes signal in control samples, confirming antibody specificity. Scale bars = 50 μm.

Supplementary Figure 3

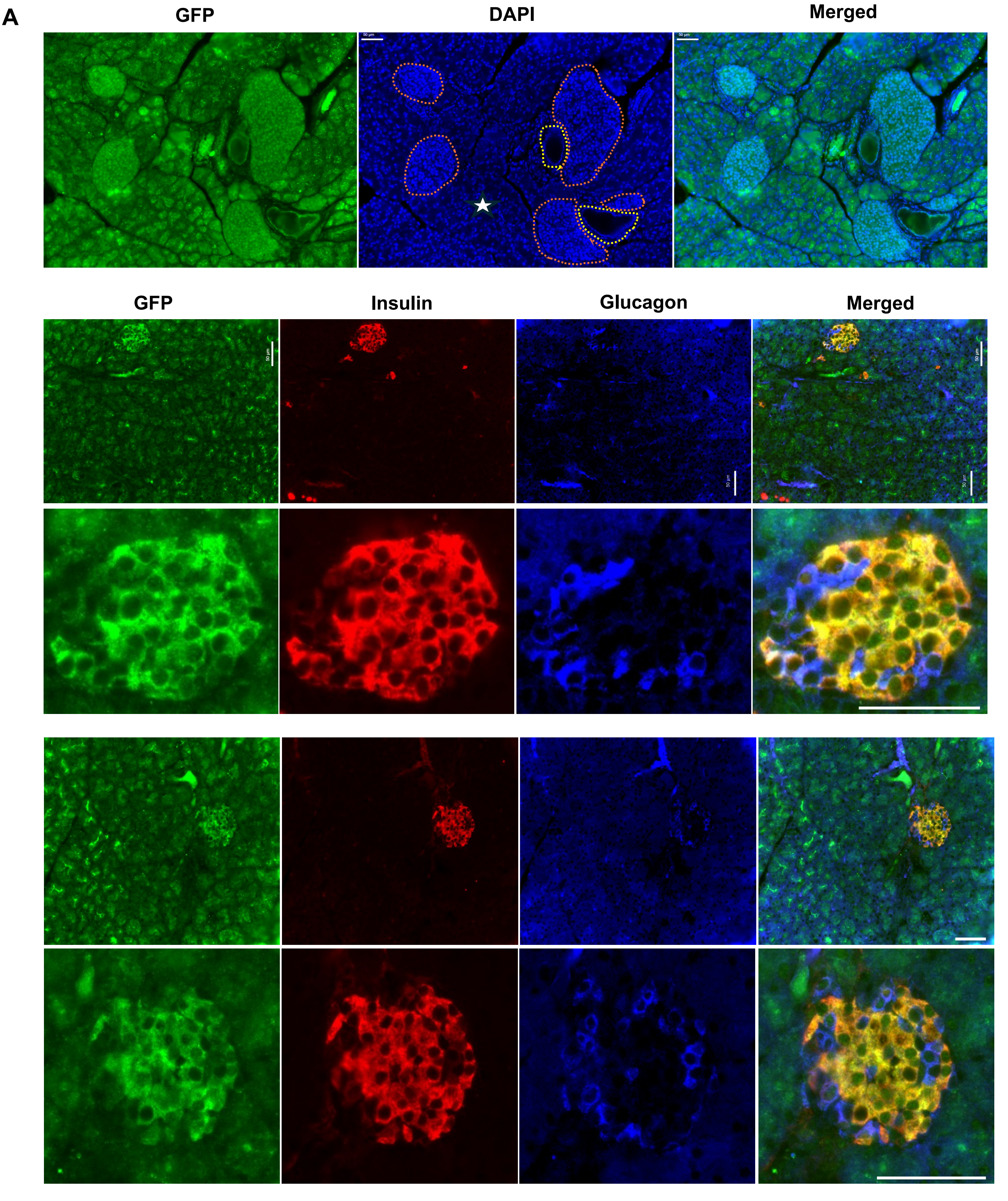

Supplementary Figure 3

B

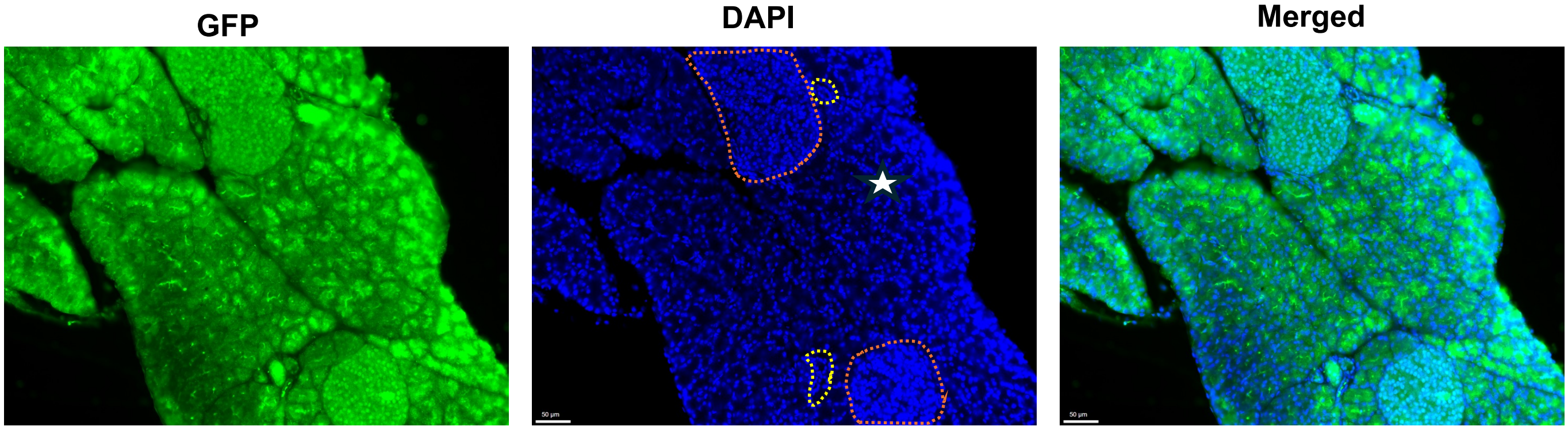

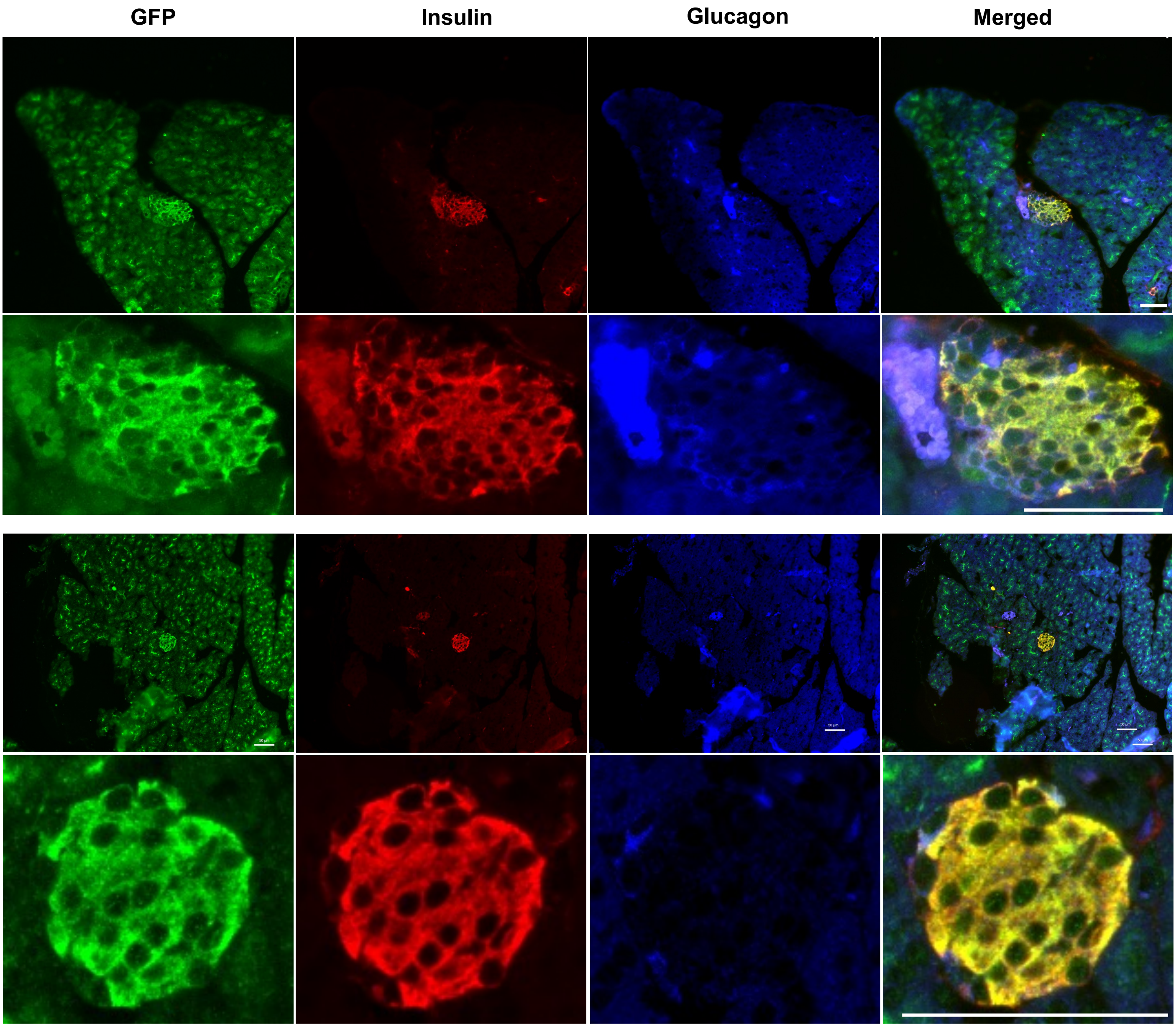

Supplementary Figure 3

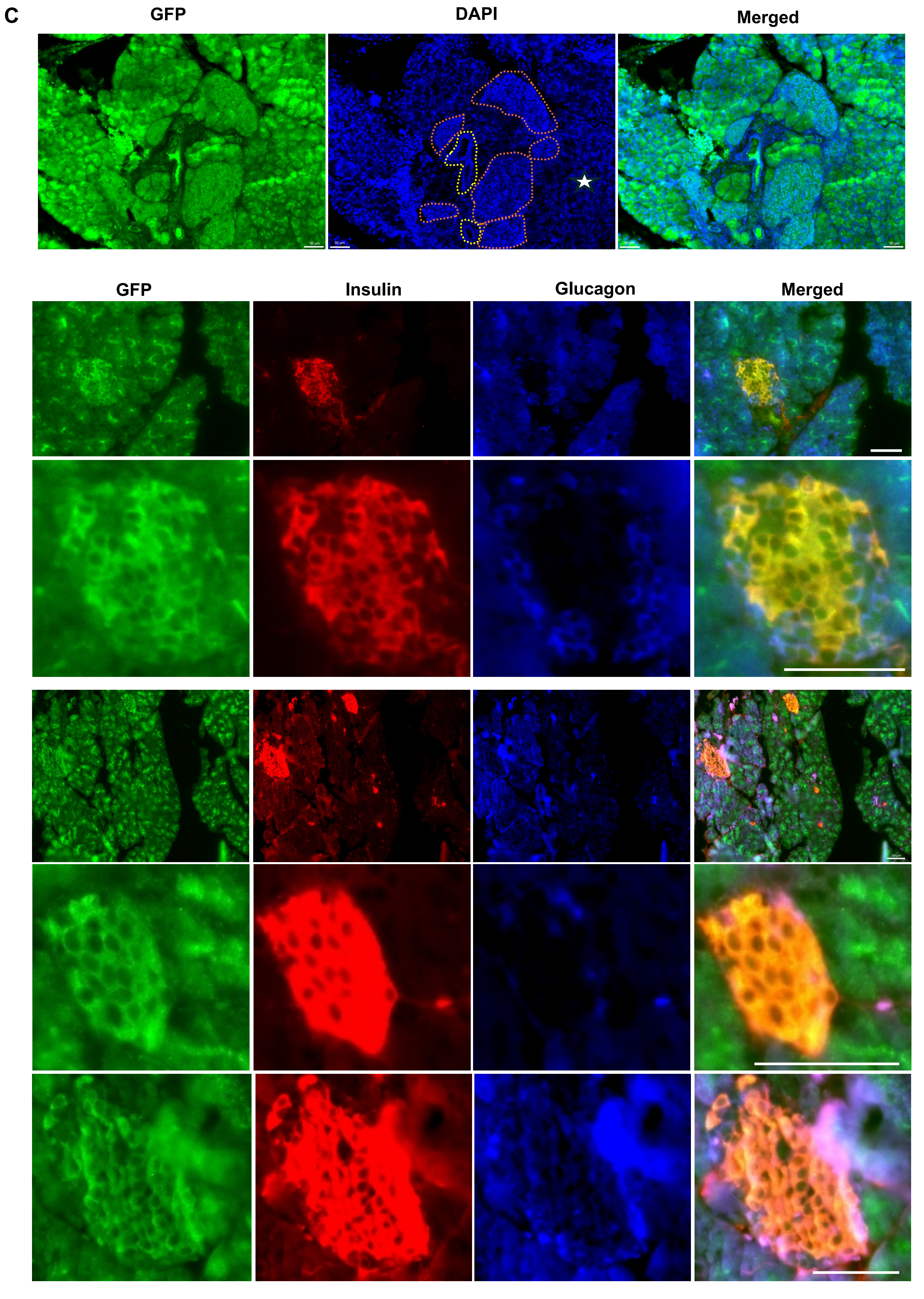

Supplementary Figure 3

D

GFP | DAPI | Merged

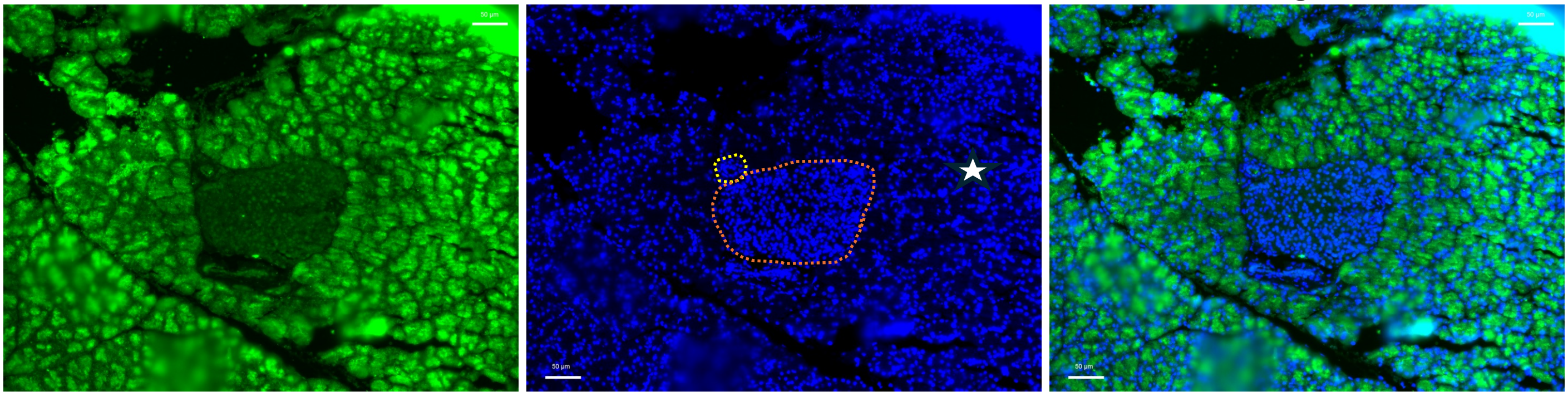

E

GFP | DAPI | Merged

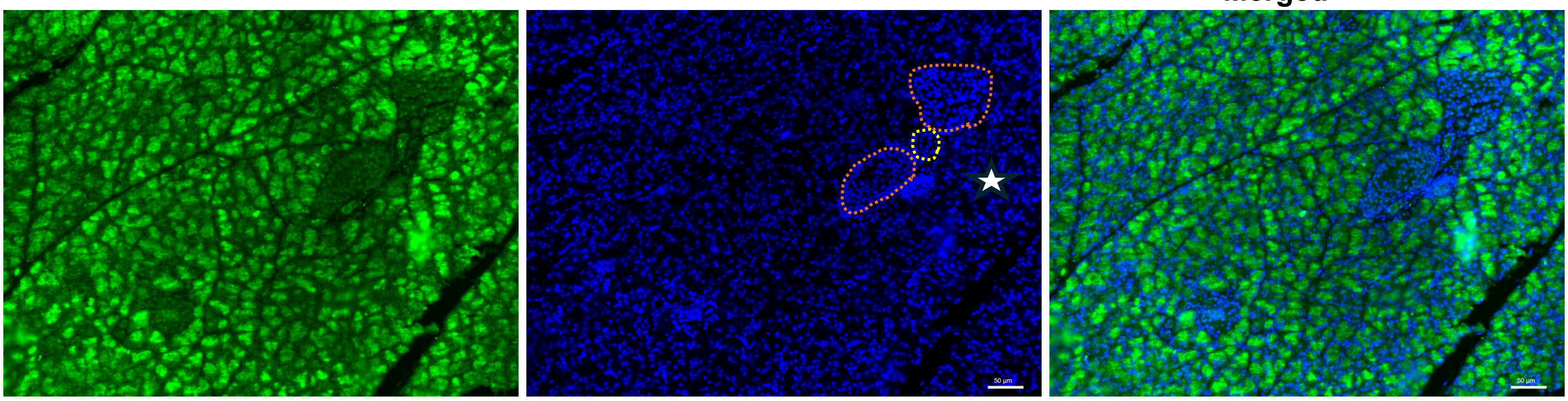

Supplementary Figure 3 | GFP immunoreactivity in individual pancreatic samples. Representative immunofluorescence images showing the distribution of GFP-positive cells in five individual pancreatic samples. **(A–C)** Samples 1–3, respectively. In each panel, the first row shows GFP immunostaining, DAPI nuclear counterstaining, and the merged image from left to right. The second row shows low-magnification images of GFP, insulin, and glucagon immunostaining, followed by the merged image. The third row shows higher-magnification views of the islet shown in the second row, presented as GFP, insulin, glucagon, and merged images from left to right. The fourth row shows low-magnification images of a second islet region, presented as GFP, insulin, glucagon, and merged images from left to right. The fifth row shows higher-magnification views of the islet shown in the fourth row, presented in the same order. In **(C)**, the sixth row shows an additional higher-magnification view of another islet within the region shown in the fourth row, with GFP, insulin, glucagon, and merged images presented from left to right. **(D, E)** Samples 4 and 5, respectively, showing GFP immunostaining, DAPI nuclear counterstaining, and merged images from left to right. Samples 1–3 exhibited GFP immunoreactivity in both endocrine islets and exocrine pancreatic compartments, whereas samples 4 and 5 exhibited GFP immunoreactivity in the exocrine compartment without detectable GFP signal in endocrine islets. Samples 6–8 are not shown because GFP-positive endocrine or exocrine pancreatic compartments were not detected; their endpoint classification is provided in Supplementary Table 2. Orange dotted lines outline islet regions, yellow dotted lines outline ductal regions, and white asterisks indicate acinar exocrine regions. Scale bars, 50 µm.

Supplementary Figure 4

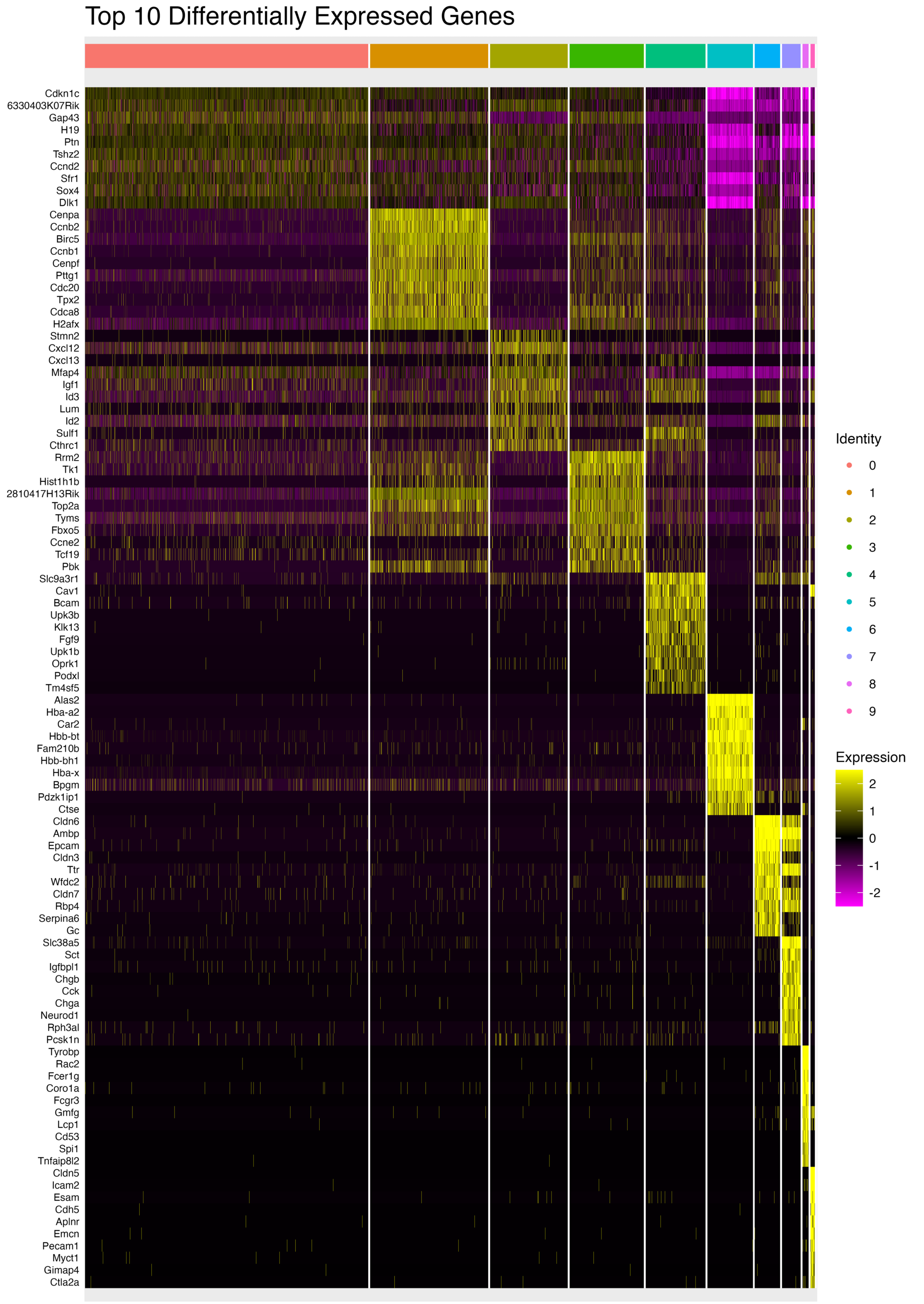

Supplementary Figure 4 | Top differentially expressed genes per cluster in the GSM2699156 dataset. Heatmap showing the top differentially expressed genes identified for each cluster following unsupervised clustering of embryonic mouse pancreas scRNA-seq data (GSM2699156). Expression values are scaled (z-score) across cells. Distinct expression patterns confirm clear transcriptional separation among the 10 clusters.

Supplementary Figure 5

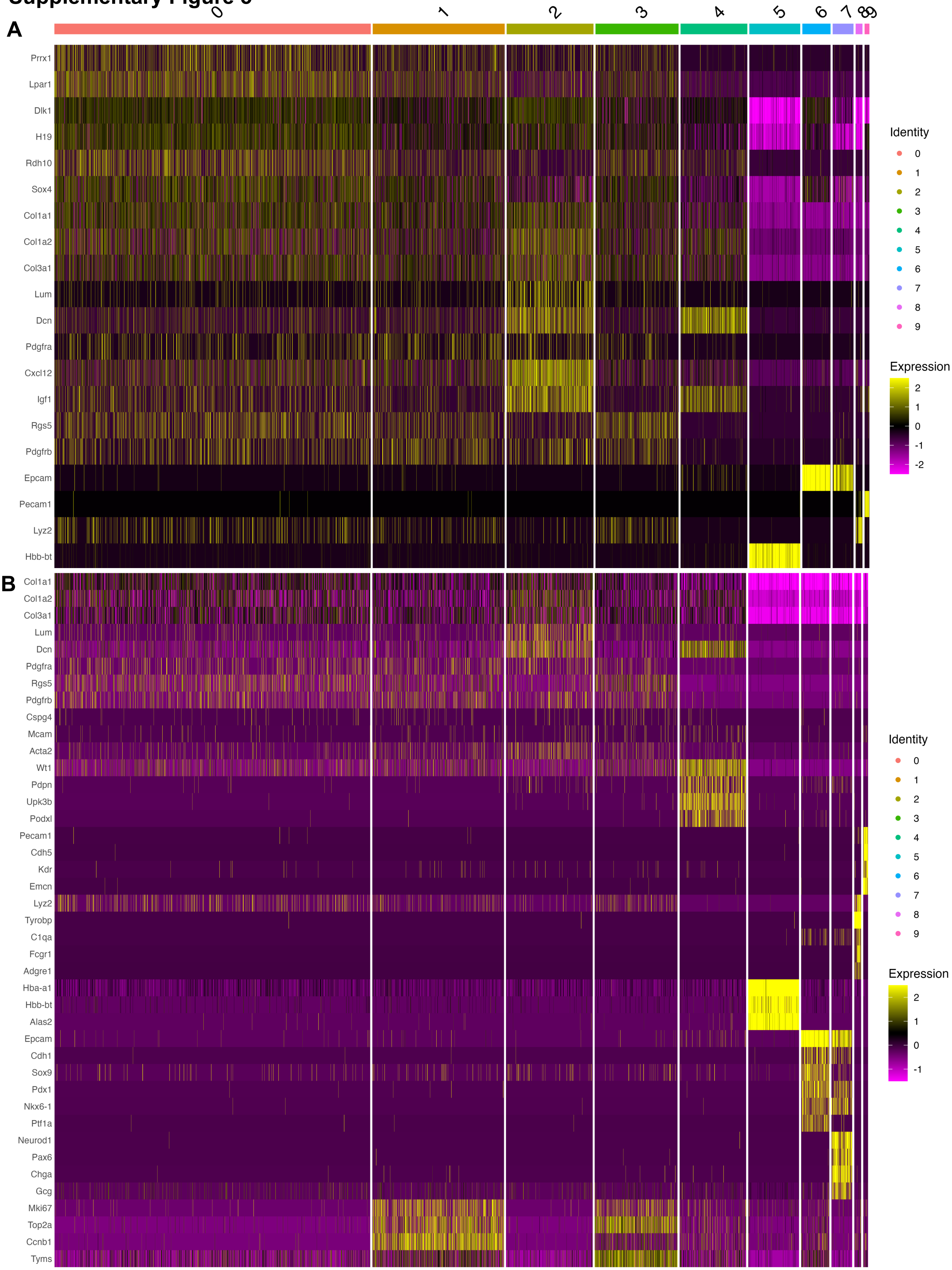

Supplementary Figure 5 | Lineage marker validation and epithelial/progenitor marker expression in GSM2699156 clusters.

(A) Heatmap showing expression of core lineage markers used for cluster annotation. (B) Heatmap showing epithelial and pancreatic progenitor markers used for cluster resolution, supporting identification of pancreatic epithelial and endocrine progenitor populations.

Supplementary Figure 6

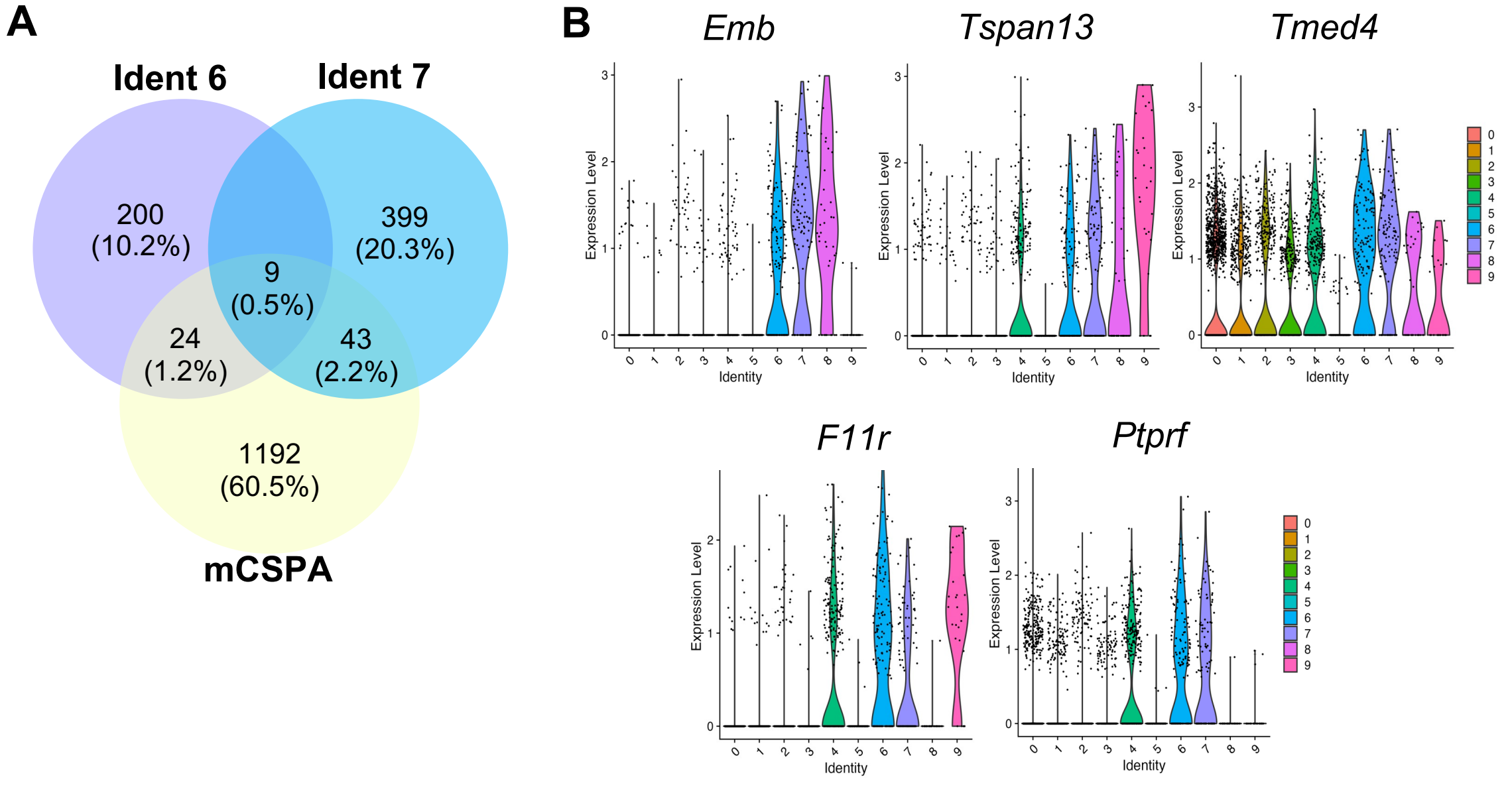

Supplementary Figure 6 | Identification of candidate surface markers enriched in Pdx1-expressing epithelial clusters.

(A) Venn diagram showing overlap between genes enriched in epithelial clusters 6 and 7 (avg-$log_2FC > 1$, adjusted $P < 0.001$) and surface proteins listed in the Cell Surface Protein Atlas (mCSPA), identifying candidate surface markers. (B) Violin plots showing expression of selected candidates (*Emb, Tspan13, Tmed4, F11r, and Ptprf*) across clusters. These genes show broader expression across multiple cell populations compared with the epithelial-restricted markers presented in Fig. 3.

Supplementary Figure 7

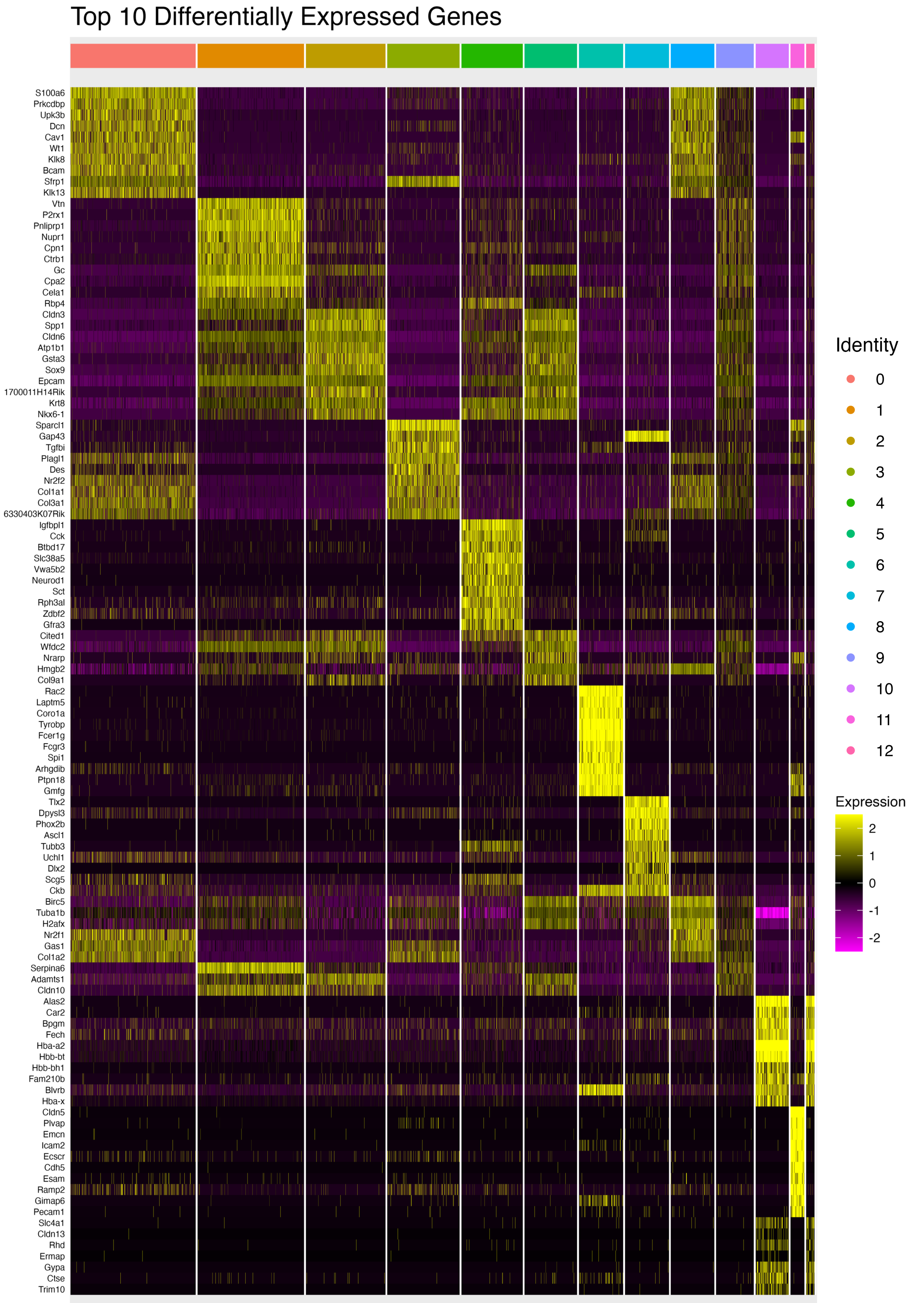

Supplementary Figure 7 | Top differentially expressed genes across clusters in the GSM3140915 dataset. Heatmap showing the top differentially expressed genes per cluster following unsupervised clustering of E12.5 mouse pancreas scRNA-seq data (GSM3140915). Expression values are scaled (z-score) across cells. Distinct expression patterns confirm clear transcriptional separation among the 13 identified clusters.

Supplementary Figure 8

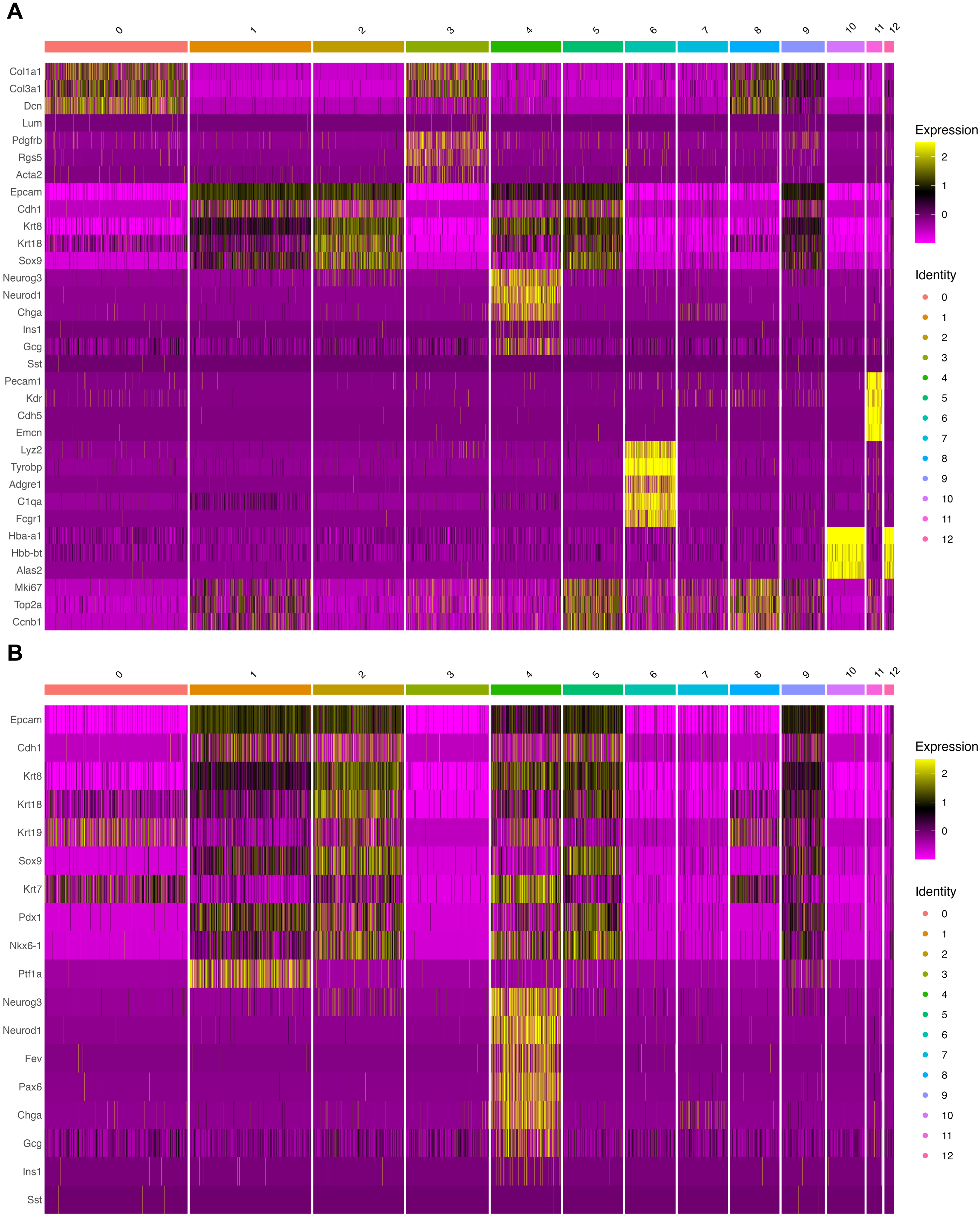

Supplementary Figure 8 | Lineage marker–based annotation of GSM3140915 clusters.
(A) Heatmap showing expression of core lineage markers used for cluster annotation.
(B) Heatmap showing epithelial and pancreatic progenitor markers used to refine epithelial cluster identity.

Supplementary Figure 9

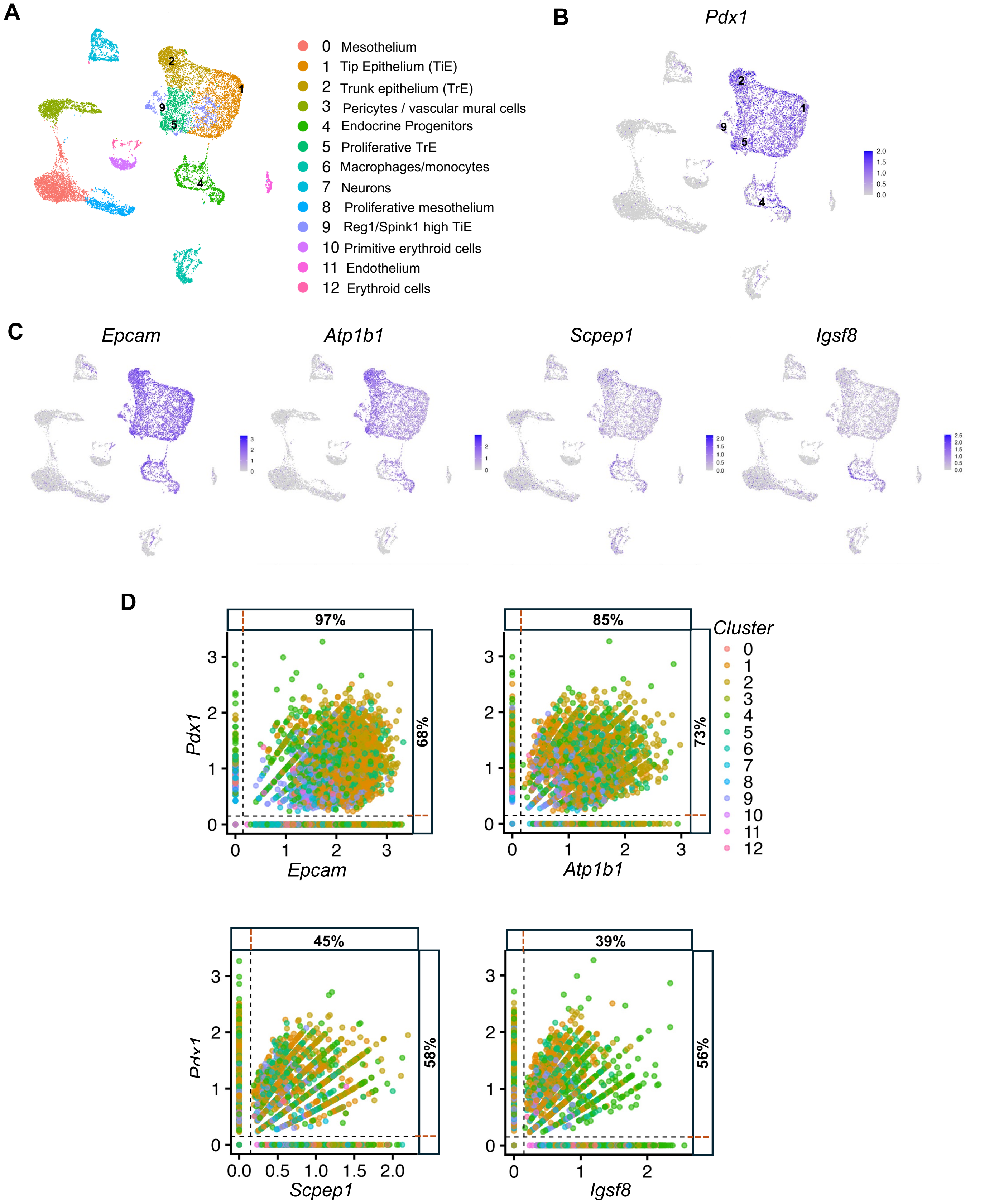

Supplementary Figure 9 | Validation of candidate surface markers in an epithelial-enriched E12.5 pancreas scRNA-seq dataset (GSM3140915). (A) UMAP visualization showing cluster annotation of the epithelial-enriched dataset. (B) UMAP feature plot showing distribution of *Pdx1* expression across clusters. (C) UMAP feature plots showing expression of candidate surface markers (*Epcam, Atp1b1, Scpep1, and Igsf8*). (D) Pseudo-flow-cytometry plots comparing normalized expression of each candidate marker with *Pdx1*. Upper percentages indicate the marker-positive fraction among *Pdx1*-positive cells for each candidate marker; marker positivity was defined as normalized expression >0.15. *Epcam* showed the highest coverage among the tested candidate markers. Percentages at right indicate the Pdx1-positive fraction among marker-positive cells for each candidate marker.

Supplementary Figure 10

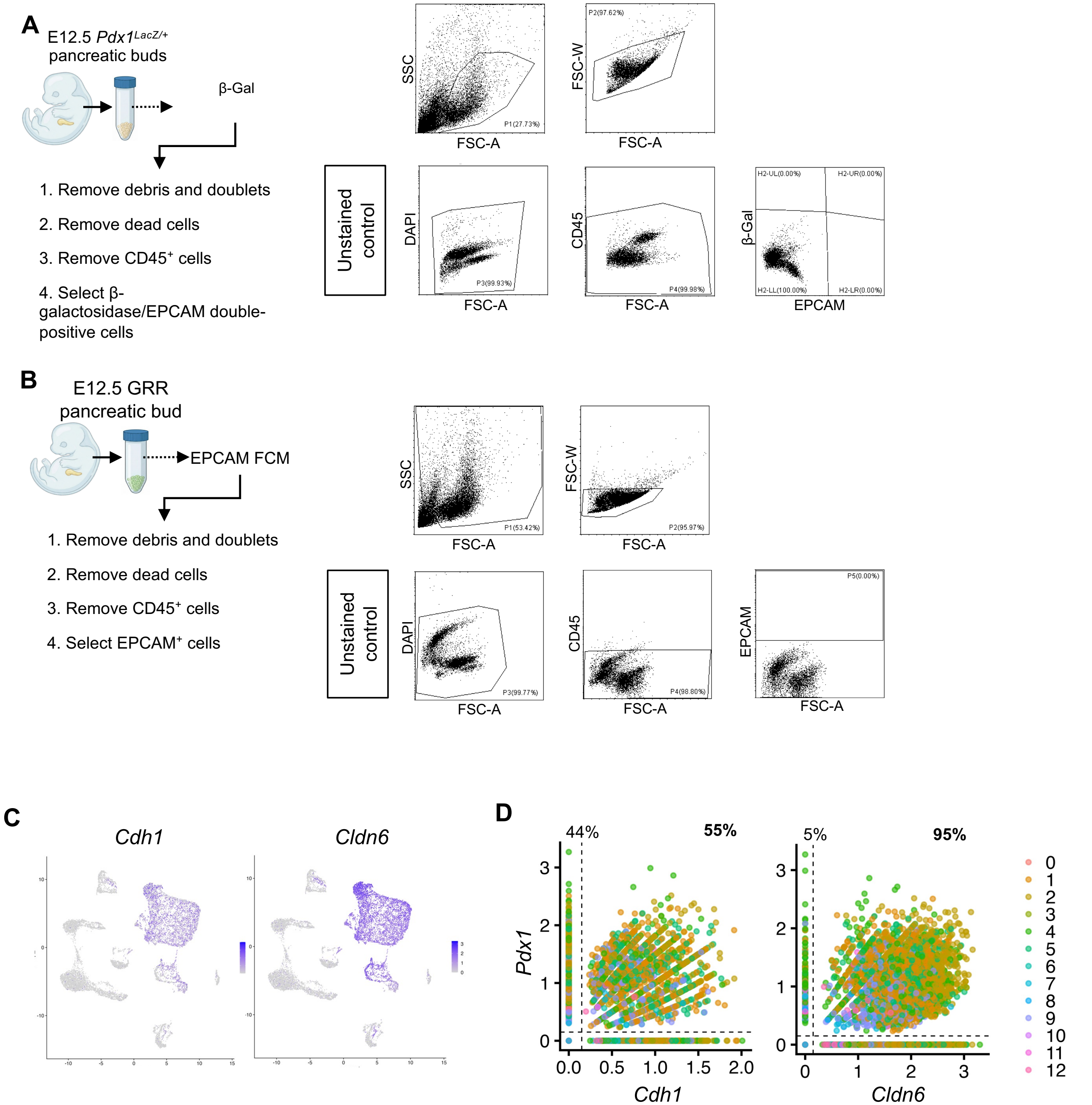

Supplementary Figure 10 | Experimental validation of EPCAM and comparison with Cdh1 and Cldn6. (A) Flow-cytometry gating strategy for detection of β-galactosidase and EPCAM in dissociated E12.5 *Pdx1*$^{LacZ/+}$ pancreatic buds using Spider-Gal and anti-EPCAM staining. (B) Flow-cytometry gating strategy for detection of EPCAM^{+} cells in dissociated E12.5 GRR pancreatic buds. (C) UMAP feature plots showing expression of *Cdh1* and *Cldn6* in the epithelial-enriched GSM3140915 dataset. (D) Pseudo-flow-cytometry plots showing normalized expression of *Pdx1* versus *Cdh1* or *Cldn6*. Percentages indicate the fraction of *Pdx1*$^{+}$ cells expressing each marker, showing lower coverage by *Cdh1* (~55%) and higher overlap for *Cldn6* (~95%).
